# Genetics of mirror movements identifies a multifunctional complex required for Netrin-1 guidance and lateralization of motor control

**DOI:** 10.1126/sciadv.add5501

**Published:** 2023-05-12

**Authors:** Sabrina Schlienger, Patricia T. Yam, Nursen Balekoglu, Hugo Ducuing, Jean-Francois Michaud, Shirin Makihara, Daniel K. Kramer, Baoyu Chen, Alfonso Fasano, Alfredo Berardelli, Fadi F. Hamdan, Guy A. Rouleau, Myriam Srour, Frederic Charron

**Affiliations:** ^1^ Montreal Clinical Research Institute (IRCM), 110 Pine Avenue West, Montreal, QC H2W 1R7, Canada.; ^2^ Department of Anatomy and Cell Biology, Division of Experimental Medicine, McGill University, Montreal, QC H3A 0G4, Canada.; ^3^ Integrated Program in Neuroscience, McGill University, Montreal, QC H3A 2B4, Canada.; ^4^ Roy J. Carver Department of Biochemistry, Biophysics and Molecular Biology, Iowa State University, Ames, IA 50011, USA.; ^5^ Edmond J. Safra Program in Parkinson's Disease, Morton and Gloria Shulman Movement Disorders Clinic, Toronto Western Hospital, UHN, Toronto, ON, Canada.; ^6^ Division of Neurology, University of Toronto, Toronto, ON, Canada.; ^7^ Krembil Brain Institute, Toronto, ON, Canada.; ^8^ IRCCS Neuromed, Pozzilli (IS), Italy.; ^9^ Department of Human Neurosciences, Sapienza University of Rome, Rome, Italy.; ^10^ Division of Medical Genetics, Department of Pediatrics, CHU Sainte-Justine and University of Montreal, Montreal, QC H3T1C5, Canada.; ^11^ Department of Human Genetics, Montreal Neurological Institute and Hospital, McGill University, Montreal, QC, Canada.; ^12^ Department of Neurology and Neurosurgery, McGill University, Montreal, QC H3A 2B4, Canada.; ^13^ Department of Pediatrics, Division of Pediatric Neurology, McGill University, Montreal, QC H4A 3J1, Canada.; ^14^ McGill University Health Center Research Institute, Montreal, QC H4A 3J1, Canada.; ^15^ Department of Medicine, University of Montreal, Montreal, QC H3T 1J4, Canada.

## Abstract

Mirror movements (MM) disorder is characterized by involuntary movements on one side of the body that mirror intentional movements on the opposite side. We performed genetic characterization of a family with autosomal dominant MM and identified *ARHGEF7*, a RhoGEF, as a candidate MM gene. We found that Arhgef7 and its partner Git1 bind directly to Dcc. Dcc is the receptor for Netrin-1, an axon guidance cue that attracts commissural axons to the midline, promoting the midline crossing of axon tracts. We show that Arhgef7 and Git1 are required for Netrin-1–mediated axon guidance and act as a multifunctional effector complex. Arhgef7/Git1 activates Rac1 and Cdc42 and inhibits Arf1 downstream of Netrin-1. Furthermore, Arhgef7/Git1, via Arf1, mediates the Netrin-1–induced increase in cell surface Dcc. Mice heterozygous for *Arhgef7* have defects in commissural axon trajectories and increased symmetrical paw placements during skilled walking, a MM-like phenotype. Thus, we have delineated how *ARHGEF7* mutation causes MM.

## Teaser

ARHGEF7 is mutated in mirror movements and is a component of a multifunctional complex required for Netrin-1/DCC axon guidance.

## INTRODUCTION

Congenital mirror movements (MM) is a neurological condition in which affected individuals have involuntary movement of a body part that mirrors the intentional movement of the contralateral homologous body part (*1*). MM predominate in the upper limbs, can be associated with pain in the upper limbs during sustained manual activities, and impair the ability of affected individuals to perform tasks requiring skilled bimanual coordination (*2*). MM result from functional and structural abnormalities in lateralized motor control, including abnormalities in commissural tracts that connect the left and right sides of the brain and spinal cord (*3*, *4*).

We previously found pathogenic variants in *DCC* (deleted in colorectal carcinoma) in individuals with congenital MM, thus establishing *DCC* as the first MM gene (*5*). *DCC* encodes the receptor for Netrin-1, an axon guidance cue that attracts axons to the midline during development, promoting the midline crossing of commissural axon tracts (*6*–*10*). Pathogenic variants in *DCC* are the most common genetic cause of congenital MM (*1*, *11*); however, pathogenic variants in other genes have also been found to cause MM: *NTN1* (encoding Netrin-1) (*12*), *RAD51* (*13*), and *DNAL4* (dynein axonemal light chain 4) (*14*). Despite these advances, the genetic, molecular, and physiological mechanisms remain poorly understood in most individuals with congenital MM.

In this study, we performed genetic characterization of a large kindred with autosomal dominant MM (*15*) and identified *ARHGEF7* as a candidate MM gene. Given that the known MM genes include *DCC* and *NTN1*, which are critical for Netrin-1/DCC axon guidance, and a third MM gene, *RAD51*, may function downstream of Netrin-1 (*16*), we hypothesized that *ARHGEF7* is also involved in Netrin-1/DCC signaling and is important for axon guidance to the midline.

In the developing spinal cord, commissural neurons send axons that project ventrally toward and subsequently across the floor plate at the ventral midline, forming axon commissures. Netrin-1 attracts commissural axons toward the ventral midline (*9*, *17*–*20*). Netrin-1 signaling activates the Rho guanosine triphosphatases (GTPases) Rac1 and Cdc42 (*21*–*24*). Rho GTPases are molecular switches that orchestrate remodeling of the actin cytoskeleton. The precise spatiotemporal control of Rho GTPases in the growth cone regulates actin cytoskeleton dynamics in response to guidance cues and drives growth cone turning (*25*, *26*).

Rho GTPases are active when bound to guanosine triphosphate (GTP) and inactive when bound to guanosine diphosphate (GDP). Guanine nucleotide exchange factors (GEFs), promote Rho GTPase activity by exchanging GDP with GTP to promote the active GTP-bound state. GEFs also integrate upstream extracellular signals from receptors to activate Rho GTPases in a context-specific manner (*27*). *ARHGEF7* encodes a RhoGEF, which regulates cell polarity, adhesion, and migration (*28*). ARHGEF7 is a GEF for Rac1 and Cdc42 (*29*). Therefore, we hypothesized that ARHGEF7 may act in Netrin-1/DCC signaling by activating Rac1 and Cdc42 downstream of Netrin-1 and that defects in ARHGEF7 disrupt Netrin-1/DCC signaling and cause defects in motor control lateralization. In this study, we show that ARHGEF7^WT^ but not ARHGEF7^mut^, an ARHGEF7 variant we found in MM individuals, directly binds to Dcc. Moreover, the ARHGEF7 binding partner Git1, an ArfGAP (adenosine diphosphate–ribosylation factor GTPase-activating protein), also directly binds to Dcc. We show that Arhgef7 activates Rac1 and Cdc42 and inhibits Arf1 downstream of Netrin-1. We also found that Arhgef7 is required for Netrin-1–mediated commissural axon guidance in vitro and for the correct trajectory of commissural axons in vivo. Mice heterozygous for *Arhgef7*, a model for the MM individuals studied herein who have a loss-of-function variant in one allele of *ARHGEF7*, displayed abnormal symmetric movements during skilled walking, recapitulating some aspects of MM. Thus, through human genetics, we have identified ARHGEF7 as a component of Netrin-1/Dcc signaling. Disruption of ARHGEF7 impairs Netrin-1/Dcc signaling and axon guidance, resulting in MM.

## RESULTS

### Familial MM is associated with a variant of *ARHGEF7*

**Fig. 1.**
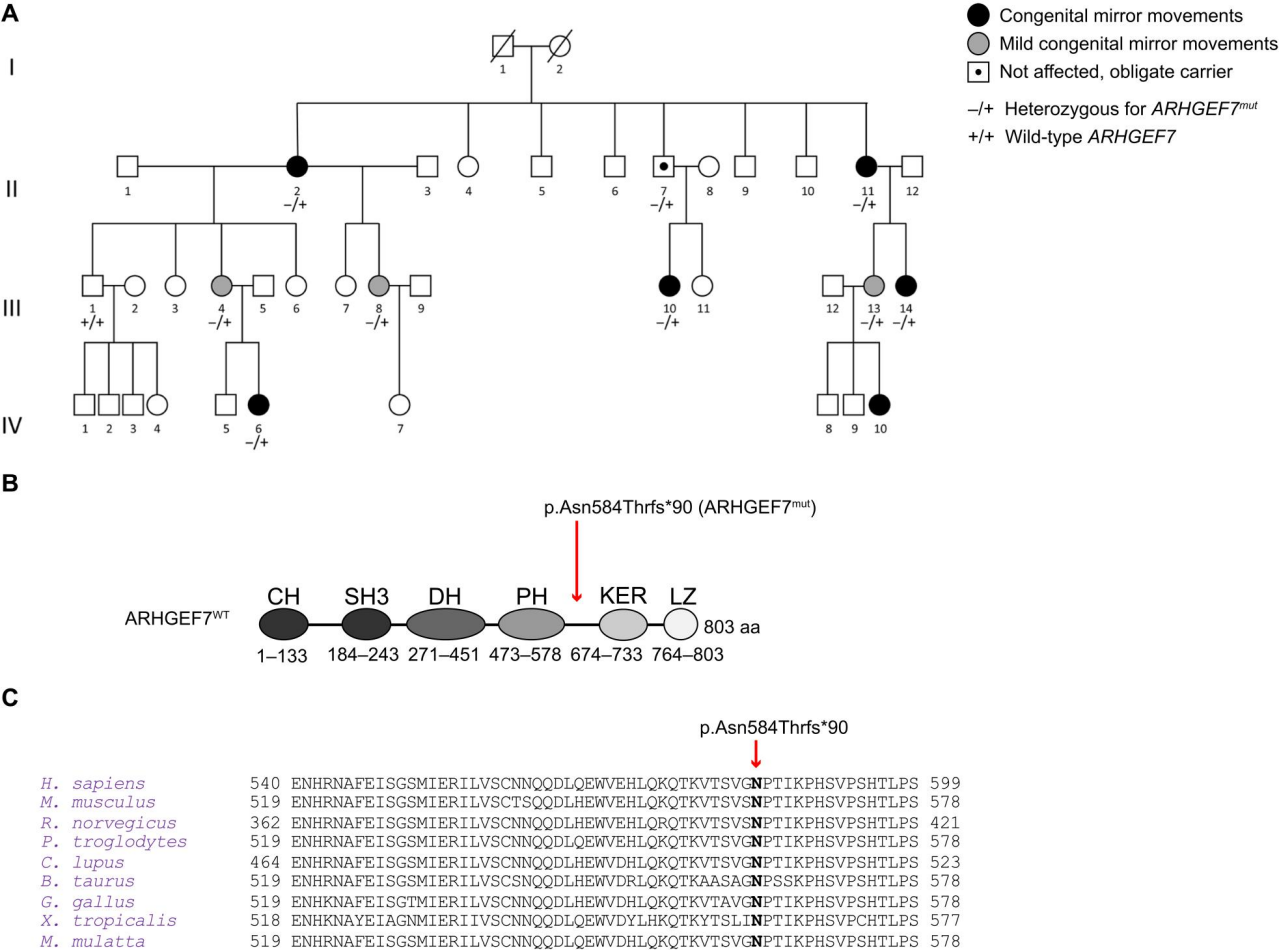
Familial MM is associated with a variant of *ARHGEF7.* (**A**) The four-generation pedigree of the MM family. Wild-type and heterozygous individuals for *ARHGEF7* p.Asn584Thrfs*90 are indicated by +/+ and −/+, respectively. (**B**) ARHGEF7 schematic. CH (calponin homology) domain (not present in adult); SH3 domain: interaction with p21-activated protein kinases 1 to 3 (PAK1,2,3); DH (Dbl homology) domain: GTPase activation; PH (pleckstrin homology) domain: membrane localization; KER (GIT-binding domain): interaction with GIT1; LZ (coiled-coil): ARHGEF7 trimerization. The position of ARHGEF7^mut^ is depicted. (**C**) Amino acid alignment of human ARHGEF7 and its predicted orthologs show conservation of the affected residue Asn^584^.

To discover previously unidentified MM genes, we examined a large four-generation family with nonsyndromic congenital MM (Fig. 1A) (*15*). Pedigree analysis suggested autosomal dominant inheritance with incomplete penetrance. Previous characterization of the affected individuals by clinical assessment, neurophysiology, and neuroimaging showed that they have a similar neurophysiological profile to individuals with MM resulting from *DCC* or *RAD51* pathogenic variants (*5*, *15*, *30*, *31*). However, sequencing of *DCC*, *RAD51*, *NTN1*, and *DNAL4* did not reveal any pathogenic variants in the affected individuals (*15*), suggesting that defects in other, yet to be identified, gene(s), cause MM in this family. Therefore, we performed whole-exome sequencing to sequence all the protein coding exons and their flanking regions of six affected members (Fig. 1A, individuals II-2, II-11, III-4, III-8, III-13, and IV-6) and one obligate carrier (II-7) of this family. We found only one predicted damaging variant in the exome data that is absent in the Genome Aggregation Database (gnomAD) and present in all seven affected individuals: a frameshift variant, c.1751_1752del, p.(Asn584Thrfs*90), in *ARHGEF7* (NM_001113511.1) (Fig. 1, A and B). *ARHGEF7* has a probability of loss-of-function intolerance score of 1, and a loss-of-function observed/expected upper bound fraction score of 0.07 in gnomAD (v2.1.1), indicating a strong intolerance to loss-of-function mutations (*32*). We then screened for presence of the *ARHGEF7* variant in three additional family members (III-1, III-10, and III-14). The variant segregated with the phenotype.

*ARHGEF7* encodes a RhoGEF known to regulate cell polarity, adhesion, and migration (*28*). The variant we identified affects Asn^584^, a residue that is highly conserved across vertebrate orthologs (Fig. 1C). The frameshift mutation at Asn^584^ is predicted to truncate the ARHGEF7 protein, resulting in the loss of its C-terminal KER and LZ domains. Given the potential ability of ARHGEF7 to regulate the cytoskeleton in axon guidance, we hypothesized that *ARHGEF7* might be a MM gene.

### Arhgef7 is required for Netrin-1–mediated commissural axon guidance

**Fig. 2.**
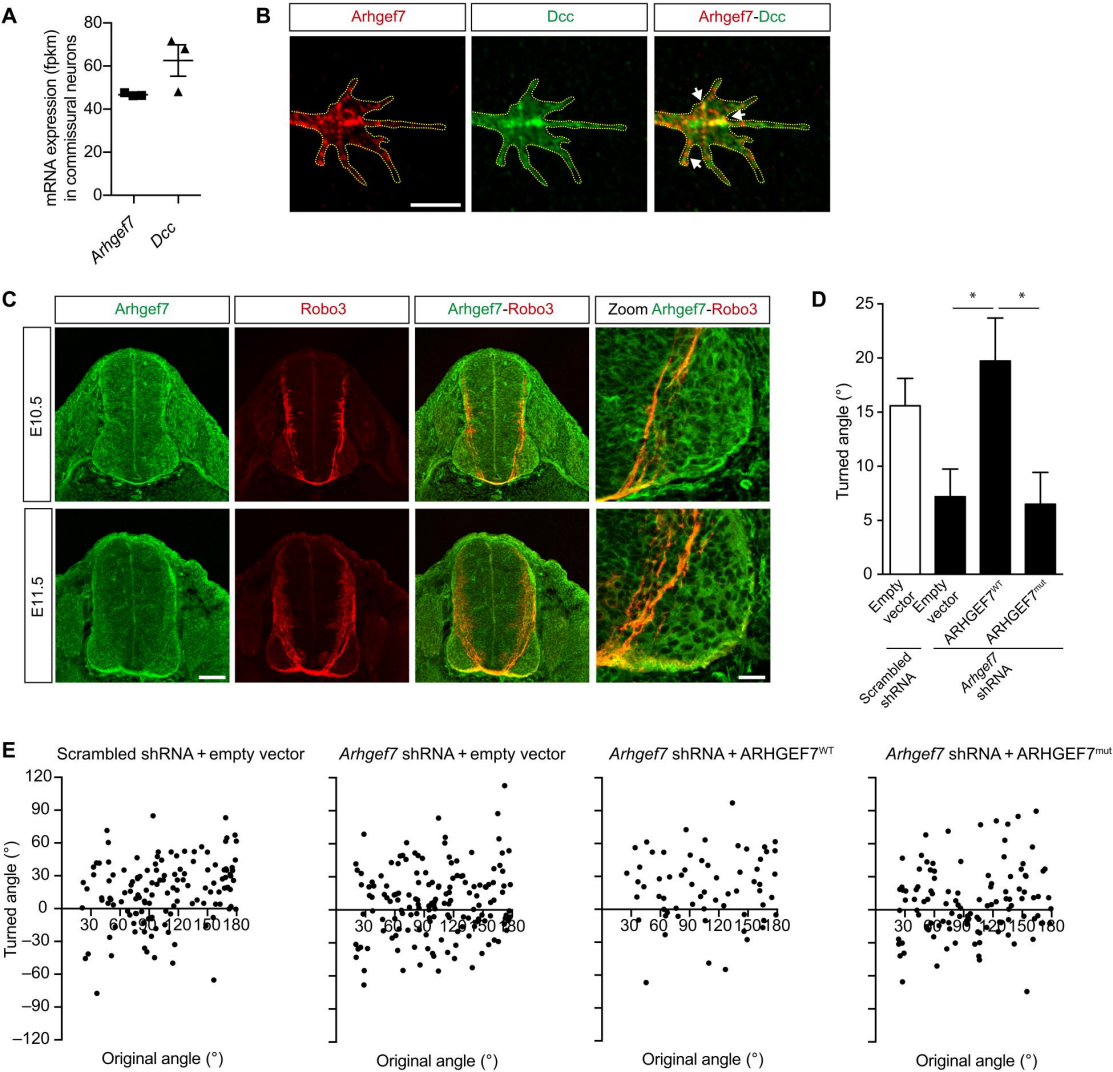
Arhgef7 is required for Netrin-1–mediated commissural axon guidance. (**A**) The mean mRNA expression, fragments per kilobase of transcript per million mapped reads (fpkm), (± SEM) of *Arhgef7* and *Dcc* in dissociated commissural neurons (*n* = 3). (**B**) Dissociated commissural neurons were fixed and immunostained for Arhgef7 and Dcc. Scale bar, 6 μm. (**C**) Mouse E10.5 and E11.5 neural tube cross sections were immunostained for Arhgef7 and Robo3, a marker of commissural neurons. Scale bar, 100 μm; zoom, 25 μm. (**D** and **E**) Commissural neurons were transfected with scrambled shRNA or shRNA against *Arhgef7*, together with empty vector, ARHGEF7^WT^ or ARHGEF7^mut^ expression vectors as indicated and then exposed to a gradient of Netrin-1 (0.1 μg/ml in the outer well) in the Dunn chamber. (D) The mean angle turned (±SEM) was quantified. (E) Scatter plots of the turned angle versus the original angle between the axons and the direction of the Netrin-1 gradient for neurons under the indicated conditions. *Arhgef7* knockdown inhibits the turning of axons up a Netrin-1 gradient, while expression of ARHGEF7^WT^ but not ARHGEF7^mut^ completely rescued Netrin-1–mediated growth cone turning. *n* = 132, 152, 63, and 115 respectively from three independent experiments. One-way analysis of variance (ANOVA), Tukey’s multiple comparison post-test, *P < 0.05. See also fig. S1.

Given the importance of Netrin-1/DCC signaling in many cases of congenital MM (*5*, *12*, *13*, *16*), we further hypothesized that ARHGEF7 functions in Netrin-1/DCC signaling, specifically in Netrin-1–mediated guidance of axons. We first determined whether *Arhgef7* is expressed in spinal cord commissural neurons, whose axons are guided by Netrin-1 to the midline (*7*, *9*). We used RNA sequencing of dissociated rat commissural neurons (*33*) and found that *Arhgef7* is expressed in commissural neurons, at a level comparable to *Dcc* (Fig. 2A). We also investigated the localization of Arhgef7 in dissociated rat commissural neurons using an anti-Arhgef7 antibody that we validated in dissociated commissural neurons knocked down for *Arhgef7* and in tissue from *Arhgef7* knockout mice (fig. S1, A to C). Immunostaining showed that Arhgef7 has a punctate distribution within growth cones and colocalizes with Dcc (Fig. 2B). Next, we investigated the Arhgef7 expression pattern in commissural neurons in the developing spinal cord of embryonic day 10.5 (E10.5) and E11.5 mouse embryos, when commissural axons project ventrally to the midline. We found that Arhgef7 is widely expressed throughout the spinal cord and that Arhgef7 colocalizes with Robo3, a marker for commissural neurons (Fig. 2C and fig. S1D). Arhgef7 appears to be present in pre- and postcrossing commissural axons. Together, our data indicate that Arhgef7 is expressed by commissural neurons and is present in growth cones.

We next tested whether Arhgef7 is required for Netrin-1–mediated axon guidance in vitro. We designed shRNAmir (short hairpin RNA with microRNA scaffold) against rat *Arhgef7* and verified that it was able to reduce endogenously expressed Arhgef7 by ~50% in commissural neurons (fig. S1, A, B, E, and F). To assess Netrin-1–mediated axon guidance, we used an in vitro axon turning assay where cultured commissural neurons are exposed to a gradient of Netrin-1 in a Dunn chamber, and their response to the gradient is then imaged and measured, with positive angles turned representing attraction up the gradient (*34*). When commissural neurons electroporated with control scrambled shRNAmir were exposed to a Netrin-1 gradient, their axons were attracted toward higher concentration of Netrin-1 (Fig. 2, D and E). In contrast, *Arhgef7* knockdown inhibited the ability of axons to be attracted by Netrin-1, demonstrating that Arhgef7 is required for Netrin-1–mediated attraction. We next sought to rescue the effect of *Arhgef7* knockdown on Netrin-1–mediated attraction by expressing human ARHGEF7^WT^ (which is not targeted by the rat *Arhgef7* shRNAmir). In the presence of *Arhgef7* shRNAmir, expression of ARHGEF7^WT^ completely rescued Netrin-1–mediated axon attraction, with the axons turning equally well toward Netrin-1 compared to the axons expressing scrambled short hairpin RNA (shRNA) control/empty vector (Fig. 2, D and E). This demonstrates that the effect of *Arhgef7* knockdown on inhibiting Netrin-1–mediated axon turning is not due to off-target effects. In contrast, expression of ARHGEF7^mut^ that has the human variant (c.1751_1752del, p.Asn584Thrfs*90) did not rescue Netrin-1–mediated attraction when *Arhgef7* was knocked down, with axons failing to turn toward a Netrin-1 gradient, similar to the axons with *Arhgef7* knockdown/empty vector (Fig. 2, D and E). This indicates that ARHGEF7^mut^ is a loss-of-function mutation. While *Arhgef7* knockdown decreased axon growth slightly, this was not associated with an inhibition of the turning response to Netrin-1, because axons knocked down for *Arhgef7* and expressing ARHGEF7^WT^ were able to turn to Netrin-1 equally well as axons electroporated with the control scrambled shRNA (fig. S1H). Together, our data demonstrate that ARHGEF7 is required for Netrin-1–mediated axon attraction and that ARHGEF7^mut^ cannot mediate axon attraction to Netrin-1.

### ARHGEF7^WT^ but not ARHGEF7^mut^ interacts with Dcc, and this interaction is modulated by Netrin-1

**Fig. 3.**
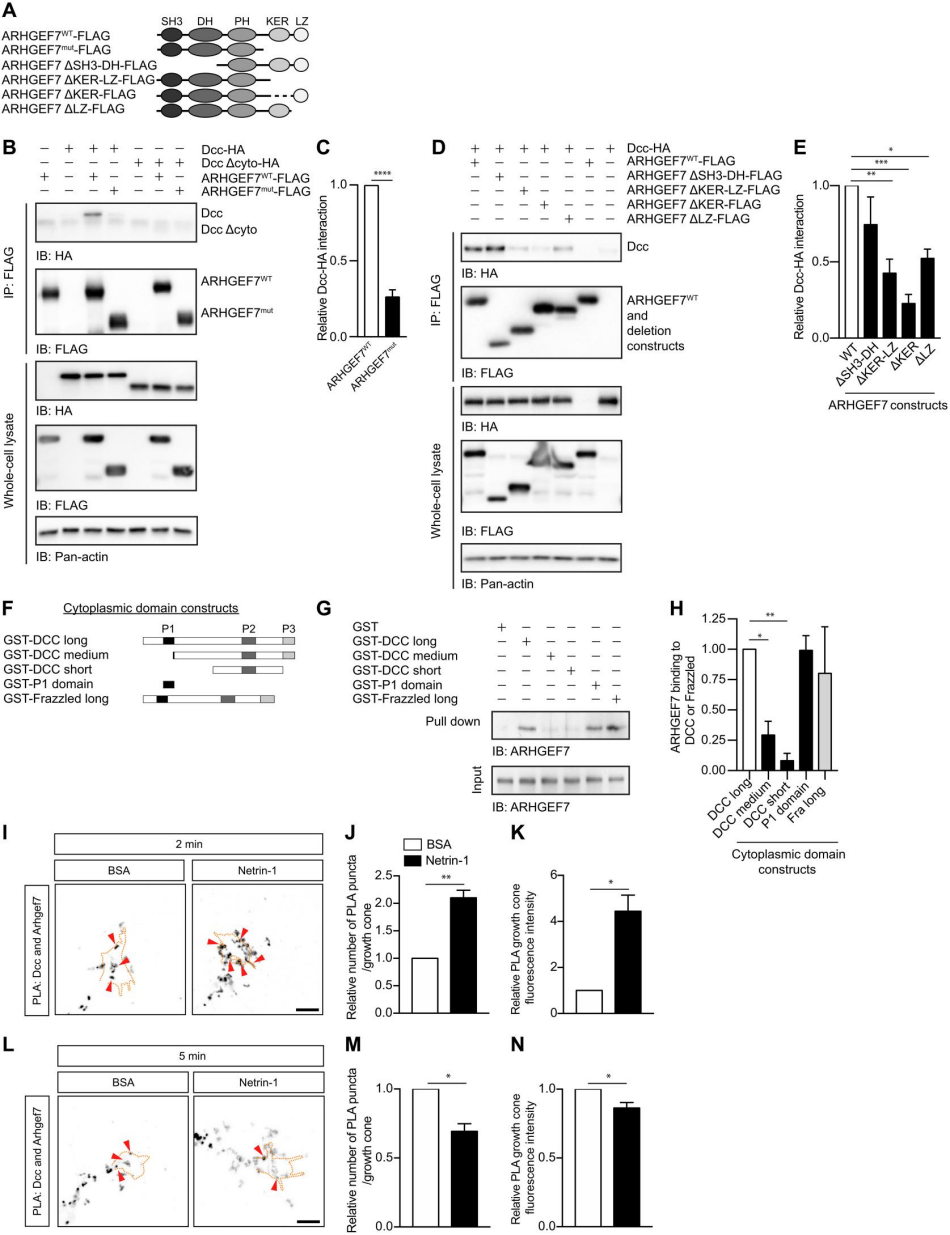
ARHGEF7^WT^, but not ARHGEF7^mut^, interacts with Dcc, and this interaction is modulated by Netrin-1. (**A**) Schematic of the ARHGEF7 constructs used. (**B** and **D**) Cos7 cells were transfected with tagged Dcc and ARHGEF7 expression vectors as indicated. The cell lysates were immunoprecipitated (IP) with an anti-Flag antibody and the immunoprecipitates analyzed by immunoblotting (IB) with the indicated antibodies. (**C**) The relative amount (mean ± SEM) of Dcc interacting with ARHGEF7^WT^ and ARHGEF7^mut^. Dcc has significantly less interaction with ARHGEF7^mut^ compared to ARHGEF7^WT^. *n* = 5, unpaired t test, ****P < 0.0001. (**E**) The relative amount (mean ± SEM) of Dcc binding to the ARHGEF7 constructs. *n* = 4, one-way ANOVA, Tukey’s multiple comparison post-test, *P < 0.05, **P < 0.01, ***P < 0.001. The interaction between ARHGEF7 and Dcc requires the KER and LZ domains. (**F**) Schematic of the DCC and Frazzled cytoplasmic domain constructs. (**G**) Purified GST fusion proteins coupled to glutathione-agarose beads were incubated with in vitro translated ARHGEF7. Pulled-down proteins were analyzed by Western blotting with an anti-ARHGEF7 antibody. ARHGEF7 directly binds to the P1 domain of DCC. (**H**) The relative binding (mean ± SEM) of ARHGEF7 to DCC/Frazzled. *n* ≥ 4, one-way ANOVA with Dunnett’s multiple comparison test. *P < 0.05, **P < 0.01. (**I** and **L**) Dissociated commissural neurons were treated with 0.1 μg/ml BSA or Netrin-1 for 2 and 5 min respectively, then fixed. The PLA assay was performed for Dcc and Arhgef7. Arrowheads indicate some examples of PLA puncta. Scale bar, 7 μm. (**J** and **M**) The relative number of PLA puncta per growth cone and (**K** and **N**) the mean (±SEM) intensity of the PLA signal in the growth cone after 2 min (J and K) and 5 min (M and N) of Netrin-1 stimulation. *n* = 4 experiments, 15 growth cones per condition, per experiment. Paired t test, *P < 0.05, **P < 0.01. See also fig. S2.

In many cases, GEFs interact with the receptor of the pathway in which they signal (*26*). Thus, to investigate how ARHGEF7 acts downstream of Netrin-1, we tested whether ARHGEF7 can interact with Dcc. We expressed epitope-tagged ARHGEF7^WT^ or ARHGEF7^mut^ (Fig. 3A) with epitope-tagged Dcc in Cos7 cells and tested whether they interact by coimmunoprecipitation (co-IP). Immunoprecipitation of ARHGEF7^WT^ resulted in the co-IP of Dcc, indicating that ARHGEF7^WT^ interacts with Dcc (Fig. 3B). However, ARHGEF7^mut^ failed to interact with Dcc (Fig. 3, B and C), suggesting that the ARHGEF7 C-terminal region containing the KER and LZ domains is important for Dcc interaction. To better characterize which domain(s) of ARHGEF7 are required for its interaction with Dcc, we expressed various ARHGEF7 deletion constructs (*35*) and tested whether Dcc could coimmunoprecipitate with them (Fig. 3, A and D). Immunoprecipitation of ARHGEF7 ΔSH3-DH resulted in the co-IP of Dcc at a similar level to ARHGEF7^WT^, indicating that the SH3 and DH domains are not required for interaction with Dcc. However, immunoprecipitation of ARHGEF7 ΔKER-LZ, ΔKER, and ΔLZ all resulted in significantly lower amounts of Dcc in the coimmunoprecipitate compared with ARHGEF7^WT^, indicating that the LZ and the KER domains are both important for the ARHGEF7/Dcc interaction (Fig. 3, D and E), consistent with our results with ARHGEF7^mut^ (Fig. 3, B and C).

To test whether the interaction between Dcc and ARHGEF7 is mediated by the cytoplasmic domain of Dcc, we expressed Dcc Δcyto, a construct lacking the cytoplasmic domain of Dcc, with ARHGEF7^WT^ or ARHGEF7^mut^. We found that Dcc Δcyto did not coimmunoprecipitate with ARHGEF7^WT^ nor ARHGEF7^mut^ (Fig. 3B), confirming that the cytoplasmic domain of Dcc is required for interaction with ARHGEF7.

Next, we determined whether the interaction between ARHGEF7 and the cytoplasmic domain of Dcc is direct. We performed a pull-down assay with the purified cytoplasmic domain of human DCC fused to glutathione *S*-transferase (GST) (GST-DCC) and in vitro translated ARHGEF7^WT^. We found that the full-length cytoplasmic domain, GST-DCC long, but not GST alone, could pull down ARHGEF7 (Fig. 3, F to H), indicating that the interaction between DCC and ARHGEF7^WT^ is direct. Then, we tested the ability of various GST-DCC cytoplasmic domain deletion constructs (Fig. 3F) to pull down ARHGEF7. Both GST-DCC medium and GCT-DCC short, which lack the P1 domain, were unable to pull down ARHGEF7. Conversely, the GST-DCC P1 domain alone was able to pull down ARHGEF7 at a similar level to GST-DCC long, indicating that the P1 domain of DCC is necessary and sufficient for the interaction between DCC and ARHGEF7. The cytoplasmic domain of Frazzled, the *Drosophila* ortholog of DCC, also directly interacts with ARHGEF7 (Fig. 3, G and H), indicating that the ARHGEF7/DCC interaction is evolutionarily conserved.

Having found that Arhgef7 binds directly to Dcc, we next tested whether endogenous Arhgef7 and Dcc also interact in commissural neurons and whether this is modulated by Netrin-1. For this, we performed a proximity ligation assay (PLA), an immunofluorescence-based assay that detects when two proteins are proximal (<40 nm) to each other and therefore likely to be interacting. In control neurons stimulated with bovine serum albumin (BSA), we detected a PLA signal between Arhgef7 and Dcc throughout the neuron, indicating that there is a basal level of Arhgef7 and Dcc interaction (Fig. 3, I and L, and fig. S2, A and B) that is independent of Netrin-1 binding, consistent with our experiments showing that the cytoplasmic domain of DCC is sufficient to bind to Arhgef7 (Fig. 3, G and H). This signal was absent when either primary antibody was omitted (fig. S2C), indicating that the signal was not due to nonspecific binding of the PLA probes. Netrin-1 stimulation for 2 min significantly increased the PLA puncta and signal intensity in the growth cone (Fig. 3, I to K, and fig. S2A), whereas stimulation for 5 min significantly reduced it (Fig. 3, L to N, and fig. S2B). This suggests that the interaction between Arhgef7 and Dcc is dynamic and regulated by Netrin-1. Together, our data demonstrate that Arhgef7 interacts with Dcc and that this interaction is modulated by Netrin-1.

### ARHGEF7^WT^, but not ARHGEF7^mut^, interacts with Git1, and they both directly bind to Dcc

**Fig. 4.**
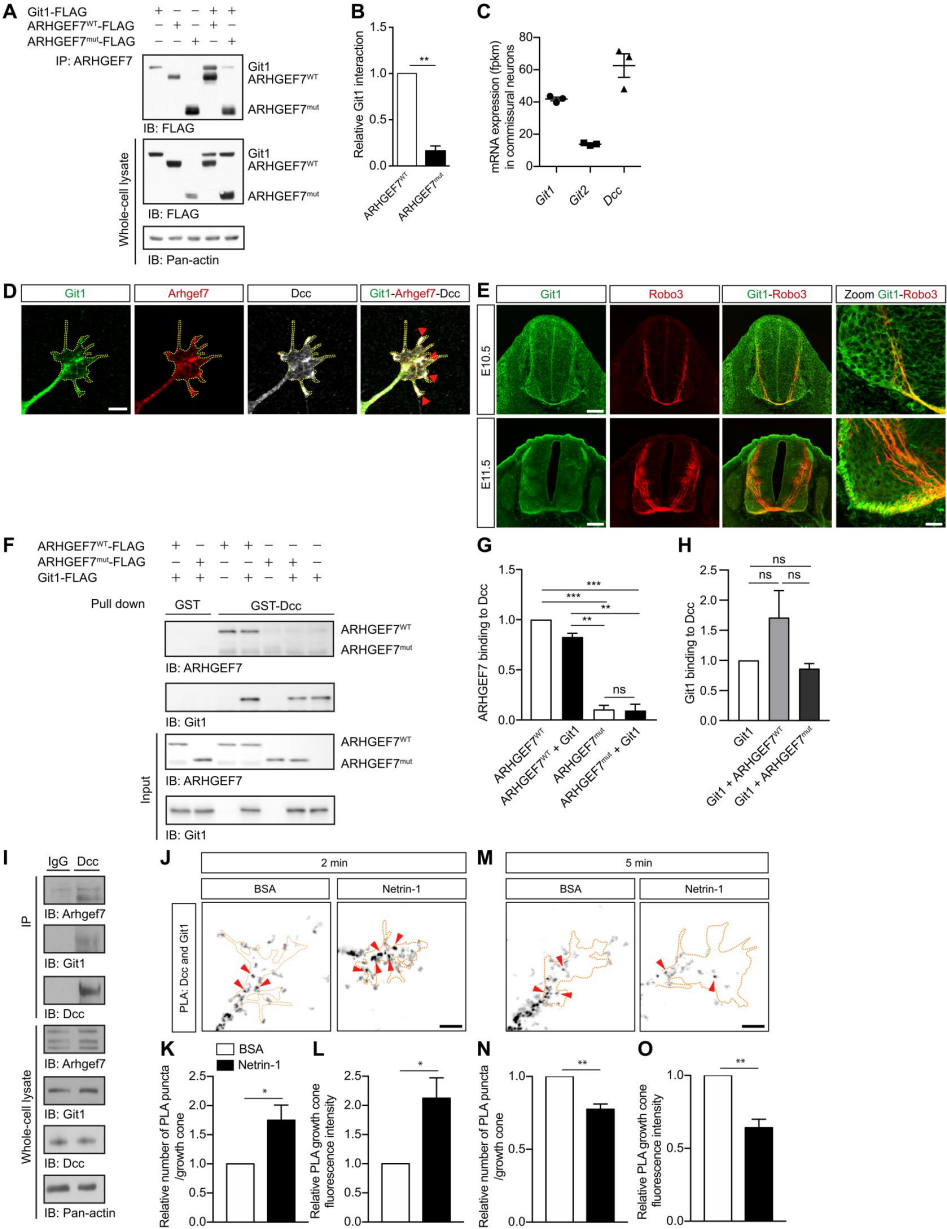
ARHGEF7^WT^, but not ARHGEF7^mut^, interacts with Git1, and they both directly bind to Dcc. (**A**) Cos7 cells were transfected with ARHGEF7 and Git1 expression vectors. Cell lysates were immunoprecipitated with an anti-ARHGEF7 antibody and analyzed by Western blotting. (**B**) Relative amount (mean ± SEM) of Git1 interacting with ARHGEF7^WT^ or ARHGEF7^mut^. Git1 has significantly less interaction with ARHGEF7^mut^ compared to ARHGEF7^WT^. *n* = 3, paired *t* test, **P < 0.01. (**C**) The mean mRNA expression (± SEM) of *Git1*, *Git2* and *Dcc* in dissociated commissural neurons (*n* = 3). (**D**) Commissural neurons immunostained for Git1, Arhgef7 and Dcc. Scale bar, 6 μm. (**E**) Mouse E10.5 and E11.5 neural tube cross sections were immunostained for Git1 and Robo3, a marker of commissural neurons. Scale bars, 100 μm; zoom, 25 μm. (**F**) Purified GST or GST-Dcc coupled to glutathione-agarose beads were incubated with in vitro translated Git1, ARHGEF7^WT^ and ARHGEF7^mut^ as indicated. Pulled-down proteins were analyzed by Western blotting. ARHGEF7^WT^ and Git1, but not ARHGEF7^mut^, directly bind to Dcc. (**G** and **H**) The relative binding (mean ± SEM) of ARHGEF7 and Git1 to Dcc. *n* = 4, two-way ANOVA with Sidak’s multiple comparison test. **P < 0.01, ***P < 0.001; one-way ANOVA with Tukey’s multiple comparison post-test. ns, not significant. (**I**) Commissural neuron lysates were immunoprecipitated with an anti-DCC antibody or IgG control and analyzed by Western blotting. (**J** and **M**) Commissural neurons were treated with BSA or Netrin-1 for 2 and 5 min and then fixed. The PLA assay was performed for Dcc and Git1. Scale bar, 7 μm. (**K** and **N**) The relative number of PLA puncta per growth cone and (**L** and **O**) the mean (±SEM) intensity of the PLA signal in the growth cone after 2 min (K and L) and 5 min (N and O) of Netrin-1 stimulation. *n* = 6 experiments, 15 growth cones per condition, per experiment. Paired *t* test, *P < 0.05, **P < 0.01. See also fig. S3.

ARHGEF7 associates with Git proteins to form an oligomeric complex (*36*). Git1 is an ArfGAP best known for its roles in promoting cell attachment, polarity, and migration (*28*). ARHGEF7 interacts with Git1 through its KER domain, also known as the Git-binding domain (*37*). Notably, ARHGEF7^mut^ lacks the KER domain (Fig. 1B) and therefore is predicted not to interact with Git1. When we co-expressed epitope-tagged Git1 with epitope-tagged ARHGEF7^WT^ or ARHGEF7^mut^, we found that Git1 coimmunoprecipitated with ARHGEF7^WT^ but not with ARHGEF7^mut^ (Fig. 4, A and B). Because ARHGEF7^mut^ is unable to interact with Git1, this raises the possibility that Git1 may be important in Netrin-1–mediated axon guidance.

Mammals express two Git proteins, *Git1* and *Git2*, and we found using RNA sequencing that both are expressed in commissural neurons (Fig. 4C). We validated an antibody against Git1 by Western blotting (WB) and immunostaining of dissociated commissural neurons knocked down for *Git1* (fig. S3, A to E). Using this antibody, we immunostained for Git1 in dissociated commissural neurons and found that Git1 has a similar punctate distribution to Arhgef7 within growth cones, colocalizes with Arhgef7, as expected, and also colocalizes with Dcc (Fig. 4D). Then, we investigated the Git1 expression pattern in the spinal cord of E10.5 and E11.5 mouse embryos. We found that Git1 is broadly expressed in the spinal cord and is present in precrossing commissural axons, which were identified by Robo3 immunostaining (Fig. 4E and fig. S3F), and also appears to be present in postcrossing commissural axons.

Given that ARHGEF7 interacts with Dcc (Fig. 3) and that ARHGEF7 also interacts with Git1 (Fig. 4, A and B) (*38*, *39*) we reasoned that Dcc might form a complex with both ARHGEF7 and Git1. To test this, we performed a pull-down assay with the purified cytoplasmic tail of rat Dcc fused to GST (GST-Dcc) and in vitro translated ARHGEF7^WT^, ARHGEF7^mut^, and Git1 proteins. As we showed previously (Fig. 3, G and H), GST-Dcc, but not GST alone, could pull down ARHGEF7^WT^. In addition, we also confirmed that ARHGEF7^mut^ is unable to bind to Dcc (Fig. 4, F and G), consistent with our co-IP results (Fig. 3, B and C). We also found that Git1 could be pulled down by GST-Dcc, indicating that the interaction between Dcc and Git1, like the interaction between Dcc and Arhgef7, is direct (Fig. 4, F and H). GST-Dcc could pull down ARHGEF7 and Git1 simultaneously, suggesting that Dcc, Arhgef7 and Git1 can form a complex. Furthermore, the binding of ARHGEF7^WT^ to Dcc is not modulated by the presence of Git1, and the binding of Git1 to Dcc is not modulated by the presence of ARHGEF7 (Fig. 4, G and H).

To test whether Dcc, Arhgef7, and Git1 can form a complex in commissural neurons, we immunoprecipitated endogenous Dcc from dissociated commissural neurons. We found that endogenous Arhgef7 and Git1 both coimmunoprecipitated with Dcc (Fig. 4I), suggesting that Dcc, Arhgef7, and Git1 can form a complex in commissural neurons.

We next used PLA to determine whether the Git1/Dcc interaction in commissural neurons is regulated by Netrin-1. In control neurons stimulated with BSA, we detected a PLA signal between Git1 and Dcc throughout the neuron, indicating that there is a basal level of Git1 and Dcc interaction (Fig. 4, J and M, and fig. S3, G and H). This signal was not due to nonspecific binding of the PLA probes (fig. S3I). Netrin-1 stimulation for 2 min significantly increased the number of PLA puncta and PLA signal intensity in the growth cone (Fig. 4, J to L, and fig. S3G), while stimulation for 5 min significantly reduced it (Fig. 4, M to O, and fig. S3H), similar to what we observed with Arhgef7 and Dcc. This demonstrates that the interaction between Git1 and Dcc follows the same Netrin-1–dependent dynamics as Arhgef7 and Dcc, consistent with Arhgef7 and Git1 acting as a complex downstream of Netrin-1 signaling.

### ARHGEF7 and Git activities are required for Netrin-1–mediated commissural axon guidance

**Fig. 5.**
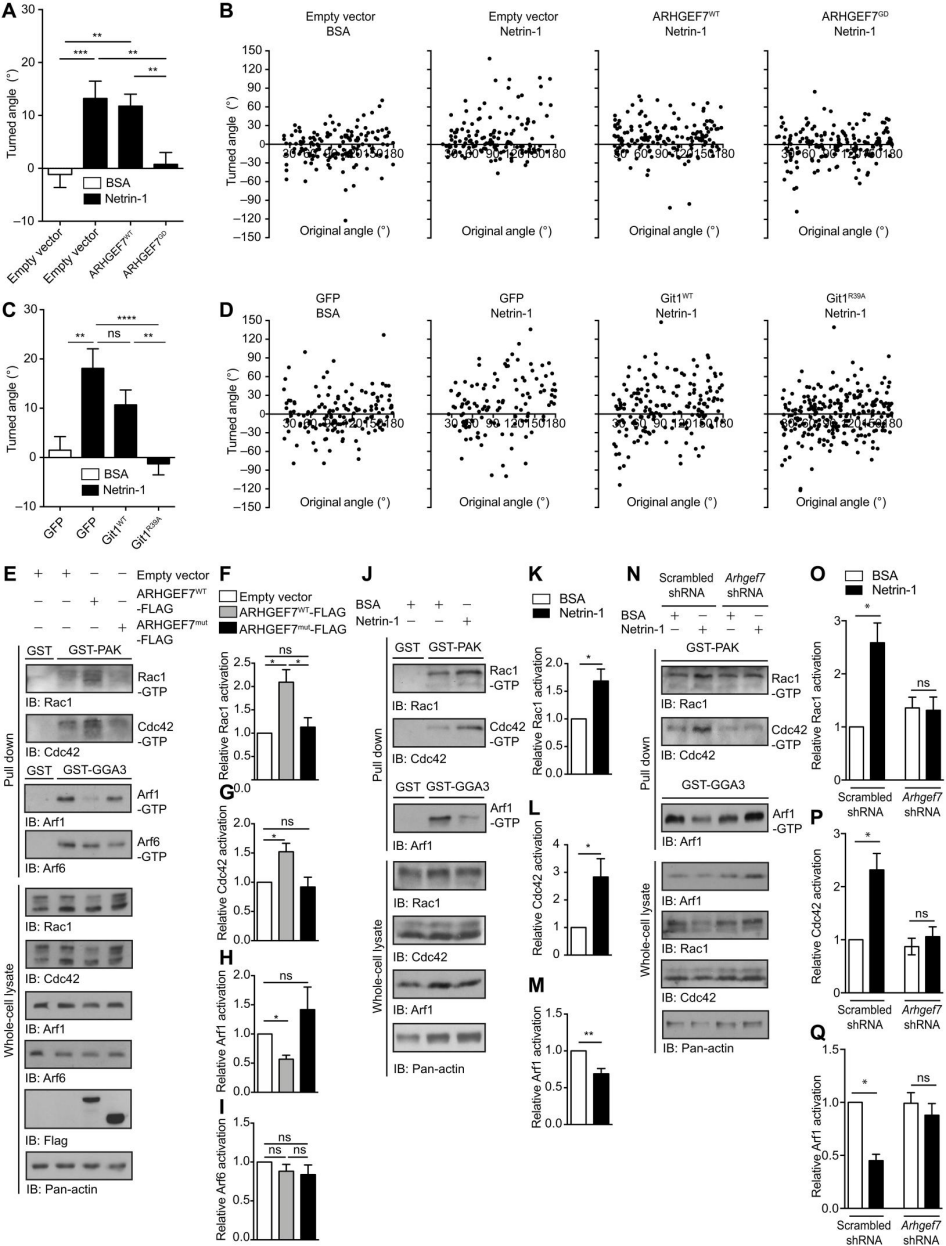
Arhgef7 is required for Netrin-1 modulation of Rac1, Cdc42 and Arf1 activity. (**A** to **D**) Commissural neurons were transfected with the indicated expression plasmids and then exposed to a Netrin-1 or BSA gradient in the Dunn chamber. (A and C) The mean turned angle (±SEM) was quantified. (B and D) Scatter plots of the turned angle versus the original angle between the axons and the direction of the gradient. (A and B) ARHGEF7^GD^ blocks axon turning toward Netrin-1. *n* = 144, 134, 161, and 166 respectively from five experiments. One-way ANOVA, Tukey’s multiple comparisons test, **P < 0.01, ***P < 0.001. (C and D) Git^R39A^ blocks axon turning toward Netrin-1. *n* = 138, 125, 176, and 234 respectively from six experiments. One-way ANOVA, Tukey’s multiple comparisons test, **P < 0.01, ****P < 0.0001. (**E**) Cos7 cells were transfected with ARHGEF7^WT-^FLAG or ARHGEF7^mut^-FLAG expression constructs. Cells were lysed and active Rac1 and Cdc42 pulled down with GST-PAK and active Arfs pulled down with GST-GGA3-coated beads. The amount of Rac1, Cdc42, Arf1 and Arf6 in pull-downs and in whole-cell lysates was detected by immunoblotting. (**F** to **I**) Relative activity of Rac1, Cdc42, Arf1 and Arf6 (mean ± SEM). ARHGEF7^WT^ but not ARHGEF7^mut^ promotes Rac1 and Cdc42 activation, and inhibits Arf1 activation. *n* = 5, one-way ANOVA, Tukey’s multiple comparison post-test, *P < 0.05. (**J**) Commissural neurons were stimulated with 0.1 μg/ml Netrin-1 or BSA for 5 min or (**N**) transfected with scrambled shRNA or *Arhgef7* shRNA and stimulated with Netrin-1 or BSA. Rac1, Cdc42, and Arf1 activation were assessed as in (E). (**K** to **M**) Relative activity of Rac1, Cdc42 and Arf1 (mean ± SEM). Netrin-1 induces Rac1 and Cdc42 activity, and inhibits Arf1 activity. *n* ≥ 8, Paired t test, *P < 0.05 **P < 0.01. (**O** to **Q**) Relative activity of Rac1, Cdc42 and Arf1 (mean ± SEM). Arhgef7 is required for Netrin-1–induced Rac1 and Cdc42 activation, and for Netrin-1–induced Arf1 inhibition. *n* = 4, one-way ANOVA, Tukey’s multiple comparison post-test, *P < 0.05. See also fig. S4.

We hypothesized that ARHGEF7 and Git1 mediate Netrin-1–induced guidance of commissural axons through activating or inactivating GTPases. We expressed ARHGEF7 and Git1 mutants that lack GEF or GAP activity respectively in commissural neurons and used the Dunn chamber axon turning assay to assess their effect on Netrin-1–mediated axon guidance. In a control BSA gradient, axons grew with no change in their trajectory, i.e., with a mean angle turned close to zero (Fig. 5, A to D). In a Netrin-1 gradient, control axons expressing either empty vector or GFP turned toward higher concentrations of Netrin-1. Expression of ARHGEF7^WT^ did not affect the ability of axons to turn toward Netrin-1. However, axons expressing ARHGEF7^GD^, which lacks GEF activity (*29*) (GEF dead, L238R/L239S), failed to turn toward Netrin-1 (Fig. 5, A and B), with no effect on axon growth (fig. S4A). Similarly, expression of Git1^R39A^, which lacks GAP activity (*40*), but not expression of Git1^WT^, also blocked the ability of axons to turn up a Netrin-1 gradient (Fig. 5, C and D), with no effect on axon growth (fig. S4B). Together, these results demonstrate that Netrin-1–mediated commissural axon guidance requires the GEF activity of ARHGEF7 and the GAP activity of Git1.

### ARHGEF7 is required for Netrin-1 modulation of Rac1, Cdc42, and Arf1 activity

ARHGEF7 and Git form a tight complex in cells and influence the stability and function of one another (*36*). They are unique among RhoGEFs and ArfGAPs in forming a constitutively associated oligomeric complex able to regulate two distinct GTP-binding protein families. ARHGEF7 activates the Rho GTPases Rac1 and Cdc42 (*29*), while Git1 inactivates the small GTPases Arf1 and Arf6 (*41*, *42*). This suggests that in ARHGEF7/Git-dependent systems, increased Rac1 and/or Cdc42 activity is coordinated with reduced Arf activity (*43*).

To test this, we expressed ARHGEF7^WT^ or ARHGEF7^mut^ in Cos7 cells and performed GTPase activation assays to measure the amount of active Rac1, Cdc42, Arf1, and Arf6 (Fig. 5E). ARHGEF7^WT^ expression increased the amount of active Rac1 and Cdc42 compared to the control (Fig. 5, E to G). Furthermore, ARHGEF7^WT^ expression decreased the amount of active Arf1 but had no significant effect on the amount of active Arf6 (Fig. 5, E, H, and I). In contrast, ARHGEF7^mut^ expression had no significant effect on the amount of active Rac1, Cdc42, nor Arf1 compared to the control (Fig. 5, E to I). These results indicate that ARHGEF7^WT^ can activate Rac1 and Cdc42 and inactivate Arf1 but that ARHGEF7^mut^ lacks this activity.

We used RNA sequencing (*33*) to confirm that *Rac1*, *Cdc42*, and *Arf1* are expressed in commissural neurons (fig. S4C). We then determined which of these GTPases are regulated by Netrin-1 in commissural neurons. We stimulated commissural neurons with Netrin-1 and found that this increased the amount of active Rac1 and Cdc42 compared to the control (Fig. 5, J to L), consistent with previous studies (*21*, *22*). Moreover, Netrin-1 stimulation decreased the amount of active Arf1 compared to the control (Fig. 5, J and M). We then tested whether *Arhgef7* knockdown affected the ability of Netrin-1 to activate Rac1 and Cdc42 or inactivate Arf1. *Arhgef7* knockdown completely suppressed the Netrin-1–induced Rac1 and Cdc42 activation and Arf1 inactivation compared to scrambled shRNAmir (Fig. 5, N and O). These results indicate that Arhgef7 is required for Netrin-1–induced Rac1 and Cdc42 activation and Arf1 inactivation in commissural neurons.

### ARHGEF7 and Git1 are required for the Netrin-1–induced increase in cell surface Dcc

**Fig. 6.**
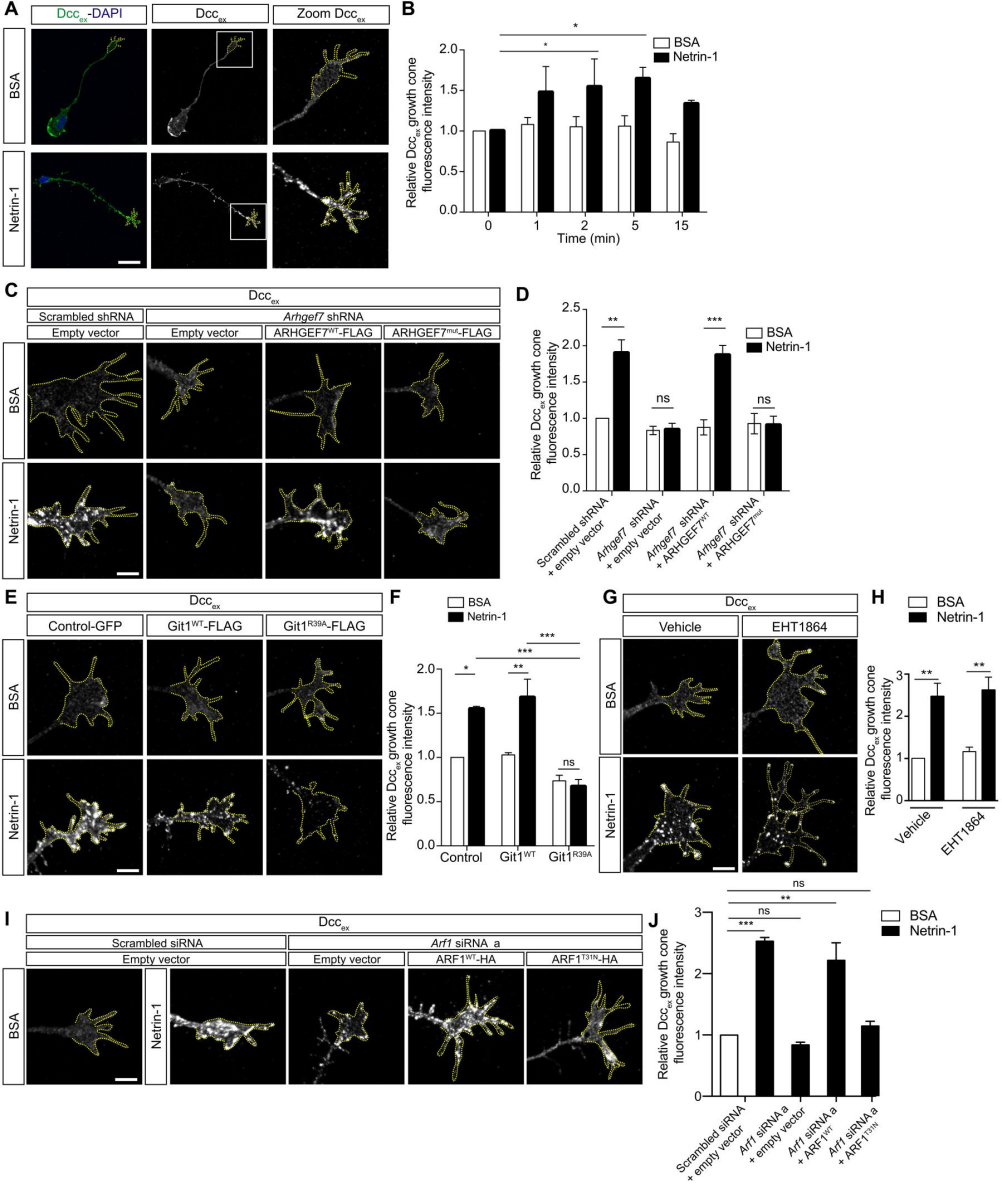
ARHGEF7 and Git are required for the Netrin-1–induced increase in cell surface Dcc. (**A**) Commissural neurons were stimulated for 1, 2, 5 and 15 min with 0.1 μg/ml Netrin-1 or BSA and fixed. Extracellular Dcc (Dcc_ex_) was detected by immunostaining under nonpermeabilizing conditions. (**B**) Mean intensity (±SEM) of Dcc_ex_ fluorescence in growth cones. Netrin-1 increases Dcc_ex_. *n* = 3 experiments, 15 growth cones per condition, per experiment. Two-way ANOVA, Sidak’s multiple comparisons test. (**C** and **E**) Commissural neurons were transfected as indicated. Cells were stimulated with 0.1 μg/ml Netrin-1 or BSA for 5 min and fixed. Dcc_ex_ was detected as in (A). (**D**) The mean intensity (±SEM) of Dcc_ex_ fluorescence in growth cones. *Arhgef7* knockdown blocks the Netrin-1–induced increase in Dcc_ex_. This is rescued by ARHGEF7^WT^ but not ARHGEF7^mut^. *n* = 4 experiments, 15 growth cones per condition, per experiment. Two-way ANOVA, Tukey’s multiple comparisons test. (**F**) Mean intensity (±SEM) of Dcc_ex_ fluorescence in growth cones. Git1^R39A^ blocks Netrin-1–induced increase in Dcc_ex_. *n* = 3 experiments, 15 growth cones per condition, per experiment. Two-way ANOVA, Sidak’s multiple comparisons test. (**G**) Commissural neurons were treated with vehicle or Rac inhibitor EHT1864 (20 μM, 2 hours). Cells were stimulated with Netrin-1 and Dcc_ex_ was detected as in (C). (**H**) Mean intensity (±SEM) of Dcc_ex_ fluorescence in growth cones. Rac inhibition does not block Netrin-1–induced increase in Dcc_ex_. *n* = 3 experiments, 15 growth cones per condition, per experiment. Two-way ANOVA, Tukey’s multiple comparisons test. (**I**) Commissural neurons were transfected as indicated. Cells were stimulated with Netrin-1 and Dcc_ex_ was detected as in (C). (**J**) Mean intensity (±SEM) of Dcc_ex_ fluorescence in growth cones. *Arf1* knockdown blocks the Netrin-1–induced increase in Dcc_ex_, and this is rescued by ARF1^WT^ but not ARF1^T31N^. *n* = 4 experiments, 15 growth cones per condition, per experiment. One-way ANOVA, Tukey’s multiple comparisons test. *P < 0.05, **P < 0.01, ***P < 0.001. Scale bars, 20 μm (A) and 7 μm (C, E, G, and I). See also figs. S5 to S8.

Netrin-1 increases the amount of cell surface Dcc, the plasma membrane localization of which is crucial for its signaling (*44*). We stimulated commissural neurons with Netrin-1 and detected cell surface Dcc (Dcc_ex_) under nonpermeabilized immunostaining conditions with an antibody directed against the extracellular domain of Dcc (fig. S5A). We found a significant increase in cell surface Dcc on the growth cones after 2 and 5 min of Netrin-1 stimulation (Fig. 6, A and B). We also detected total Dcc under permeabilized immunostaining conditions with an antibody directed against the intracellular domain of Dcc and found that there was no change in total Dcc in the growth cone after Netrin-1 stimulation (fig. S5, A to C). This indicates that the increase in cell surface Dcc was not due to a change in the total amount of Dcc, consistent with the cell surface increase resulting from a translocation of Dcc to the cell surface from an intracellular pool (*44*).

We next investigated whether Arhgef7 and Git1 are required for this increase in growth cone cell surface Dcc downstream of Netrin-1. We first knocked down *Arhgef7* in commissural neurons and verified that it had no effect on total Dcc expression (fig. S1, E and G). However, *Arhgef7* knockdown blocked the Netrin-1–induced increased cell surface Dcc observed in control scrambled shRNA neurons (Fig. 6, C and D, and fig. S5D). Thus, Arhgef7 is required for the Netrin-1–induced increase in cell surface Dcc. Expression of ARHGEF^WT^ in *Arhgef7* knocked-down neurons rescued the increase in cell surface Dcc. However, ARHGEF7^mut^ was unable to rescue the increase in cell surface Dcc (Fig. 5, C and D, and fig. S5D), consistent with ARHGEF7^mut^ being a loss-of-function mutation.

Then, to test whether Git1 is also required for the Netrin-1–induced increase in cell surface Dcc, we expressed Git1^WT^ or Git1^R39A^ in commissural neurons and confirmed that expression of Git1^WT^ or Git1^R39A^ had no effect on total Dcc expression (fig. S6, A and B). Commissural neurons expressing GFP or Git^WT^ showed an increase in cell surface Dcc after Netrin-1 stimulation. In contrast, neurons expressing Git1^R39A^ showed no increase in cell surface Dcc after Netrin-1 stimulation (Fig. 6, E and F, and fig. S6C). Together, these results demonstrate that Arhgef7 and Git1 activity are required for the Netrin-1–induced increase in cell surface Dcc at the growth cone.

We then investigated which small GTPase(s) downstream of Arhgef7/Git1 is required for the Netrin-1–induced increase in cell surface Dcc. Inhibition of Rac1 activity with 20 mM EHT1864, a well-characterized Rac1 inhibitor (*45*, *46*), for 2 hours, completely suppressed Netrin-1–induced Rac1 activation in commissural neurons (fig. S7, A and B). However, this had no effect on the Netrin-1–induced increase in cell surface Dcc at the growth cone (Fig. 6, G and H, and fig. S7C), even when Rac1 inhibition was performed overnight (fig. S7D). This implies that the increase in cell surface Dcc does not require Rac1 activity.

To ablate Arf1 activity, we used two different small interfering RNAs (siRNAs) targeted against *Arf1*. These siRNAs knocked down endogenous Arf1 in commissural neurons by >50% (fig. S8, A and B) and have no effect on total Dcc expression (fig. S8C). *Arf1* knockdown in commissural neurons blocked the Netrin-1–induced increase in cell surface Dcc observed in control neurons, demonstrating that Arf1 is required for the increase in cell surface Dcc (Fig. 6, I and J, and fig. S8, D and E). Expression of human ARF1^WT^ (which is not targeted by the rat *Arf1* siRNA) in *Arf1* knocked-down neurons rescued the effect of Netrin-1 on cell surface Dcc induction (Fig. 6, I and J, and fig. S8F), demonstrating that the effect of *Arf1* knockdown on inhibiting the Netrin-1–induced increase in cell surface Dcc is not due to off-target effects. In contrast, expression of ARF1^T31N^, a dominant-negative mutant with low affinity for both GDP and GTP (*47*, *48*), failed to rescue the increase in cell surface Dcc in *Arf1* knocked-down neurons (Fig. 6, I and J, and fig. S8F), suggesting that Arf1 activity is required for the Netrin-1–induced increase in cell surface Dcc. Because *Arf1* knockdown and decreased Arf1 activity blocked the increase in cell surface Dcc, similar to *Arhgef7* knockdown and Git1^R39A^ expression, which increase Arf1 activity, this suggests that the cycling of Arf1 between the active GTP–bound and inactive GDP–bound state is important for its function (*49*), as has been shown in other systems (*50*).

### Arhgef7 is required for normal commissural axon guidance in vivo

The *ARHGEF7* variant that we identified in the familial MM case is heterozygous. Since ARHGEF7^mut^ is a loss-of-function mutant, this suggests that the MM phenotype results from haploinsufficiency.

**Fig. 7.**
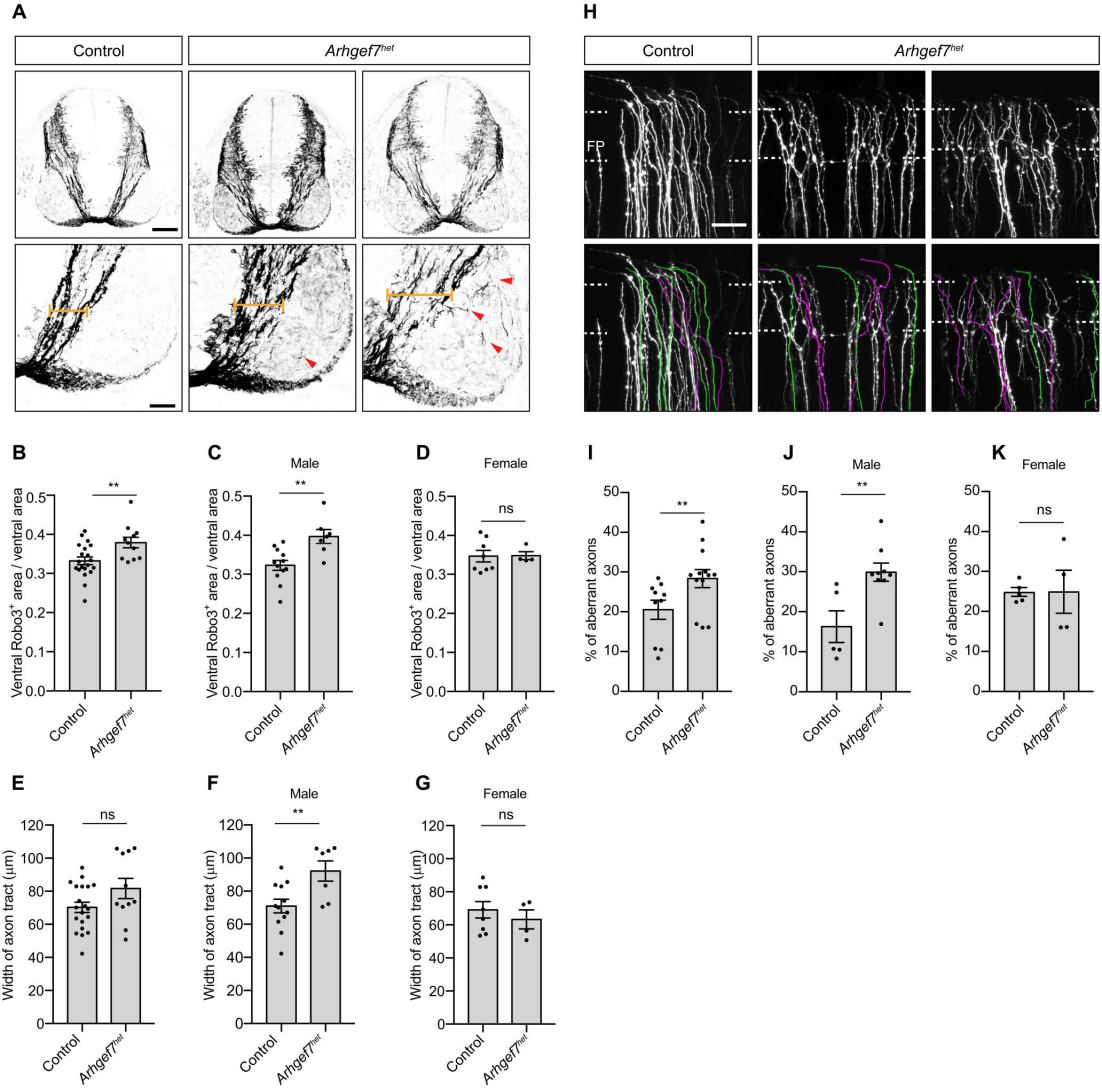
Arhgef7 is required for normal commissural axon guidance in vivo. (**A**) Robo3 immunostaining of E11.5 spinal cord cross sections of *Arhgef7^het^* mice shows that commissural axons aberrantly invade the motor column (red arrowheads) and form a wider axon tract (yellow brackets) compared to control mice. Scale bar, 100 μm; zoom, 25 μm. (**B** to **D**) Quantification of the ventral area occupied by Robo3^+^ axons relative to the area of the ventral neural tube (mean ± SEM) for all embryos (B), males (C), and females (D). Number of embryos: control *n* = 20 (12 male, 8 female), *Arhgef7^het^ n* = 11 (7 male, 4 female), ≥3 sections analyzed per embryo. Mann-Whitney test, **P < 0.01. (**E** to **G**) Quantification of the width of the commissural axon tract for all embryos (E), males (F), and females (G). Number of embryos: control n = 20, *Arhgef7^het^* n = 11, ≥3 sections analyzed per embryo. Mann-Whitney test, **P < 0.01. (**H**) DiI labeling of E13.5 open book preparations of control and *Arhgef7^het^* mice. Anterior is to the left. Representative commissural axons which cross the floor plate (FP) with a normal trajectory are pseudocolored in green. Representative commissural axons which cross the floor plate with an aberrant trajectory are pseudocolored in magenta. *Arhgef7^het^* mice have more commissural axons which cross the floor plate aberrantly compared to control mice. Scale bar, 50 μm. Dashed line, floor plate. (**I** to **K**) Quantification of the percentage of aberrant axons crossing the floor plate (mean ± SEM) for all embryos (I), males (J), and females (K). Number of embryos: control *n* = 10 [5 male (362 axons), 5 female (503 axons)], *Arhgef7^het^ n* = 13 [9 male (1062 axons), 4 female (308 axons)], one to five regions with DiI tracing analyzed per embryo. Mann-Whitney test, **P < 0.01. See also fig. S9.

*Arhgef7^−/−^* mice die embryonically at E8.5 (*51*), but heterozygous mice are viable. We used heterozygous *Arhgef7* mice, referred as *Arhgef7^het^*, to model MM individuals who carry a loss-of-function variant in one allele of *ARHGEF7*, and compared them to control mice. First, we analyzed the trajectory of commissural axons by immunostaining E11.5 spinal cord cross sections for Robo3, a marker of commissural axons. Robo3 immunostaining showed that commissural axons in *Arhgef7^het^* mice were more dispersed in the ventral spinal cord. The axon tract in *Arhgef7^het^* embryos was wider, and in some cases stray axons deviated from the main tract and invaded the motor column (Fig. 7A). To measure how dispersed the axons were, we quantified the Robo3^+^ area occupied by commissural axons in the ventral third of the spinal cord, relative to the total ventral area, and found that it was significantly larger in *Arhgef7^het^* embryos compared to control embryos (Fig. 7B). This demonstrates that loss of one allele of *Arhgef7* is sufficient to cause axon guidance defects in vivo.

To determine whether there is a sex bias in the phenotype of *Arhgef7^het^* mice, we stratified our data by sex. We found that the relative area occupied by Robo3^+^ axons in the ventral third of the neural tube was significantly higher in male *Arhgef7^het^* mice compared to control mice, but not in females (Fig. 7, C and D). We also quantified the width of the commissural axon tract. Male *Arhgef7^het^* mice also showed an increase of the width of the commissural axon tract compared to control mice, whereas female *Arhgef7^het^* mice did not (Fig. 7, E to G). To exclude the possibility that the phenotype we observed is due to changes in motor neuron specification, we measured the number of cells positive for Isl1/2, a marker for motor neurons, in control and *Arhgef7^het^* mice. We found that the number of Isl1/2+ cells in the motor column is not significantly different between control and *Arhgef7^het^* mice (fig. S9). Overall, these results demonstrate that *Arhgef7* is required for the pathfinding of commissural axons to the midline.

Defects in Netrin-1 signaling have also been associated with abnormal midline crossing of commissural axons (*18*). To analyze midline crossing in *Arhgef7^het^* embryos, we placed DiI crystals in the dorsal spinal cord and imaged “open book” preparations to visualize the trajectory of commissural axons. In control embryos, most of the axons crossed the floor plate with a straight trajectory (Fig. 7H, green axons). Some axons had an aberrant trajectory, which we defined as either the axon transiently traveling along the ipsilateral tract before entering the floor plate, the axon making strong turns within the floor plate, and/or the axon crossing the floor plate diagonally (Fig. 7H, magenta axons). We found that commissural axons in *Arhgef7^het^* embryos had more aberrantly crossing axons compared to control embryos (Fig. 7, H and I). Moreover, when we stratified the data by sex, we found that only male *Arhgef7^het^* embryos, but not female *Arhgef7^het^* embryos, had more aberrant axons compared to the control embryos (Fig. 7, J and K). Therefore, Arhgef7 is required for correct midline crossing of commissural axons. Notably, this *Arhgef7^het^* phenotype is similar to the phenotype observed when Netrin-1 is removed from the floor plate (*18*). Thus, together with our in vitro data showing that *Arhgef7* is required for commissural axons to turn toward a Netrin-1 gradient (Figs. 2, D and E, and 5, A and B), we conclude that *Arhgef7* is required for Netrin-1–mediated guidance of commissural axons in vitro and in vivo.

### *Arhgef7^het^* mice have increased symmetrical paw placements

**Fig. 8.**
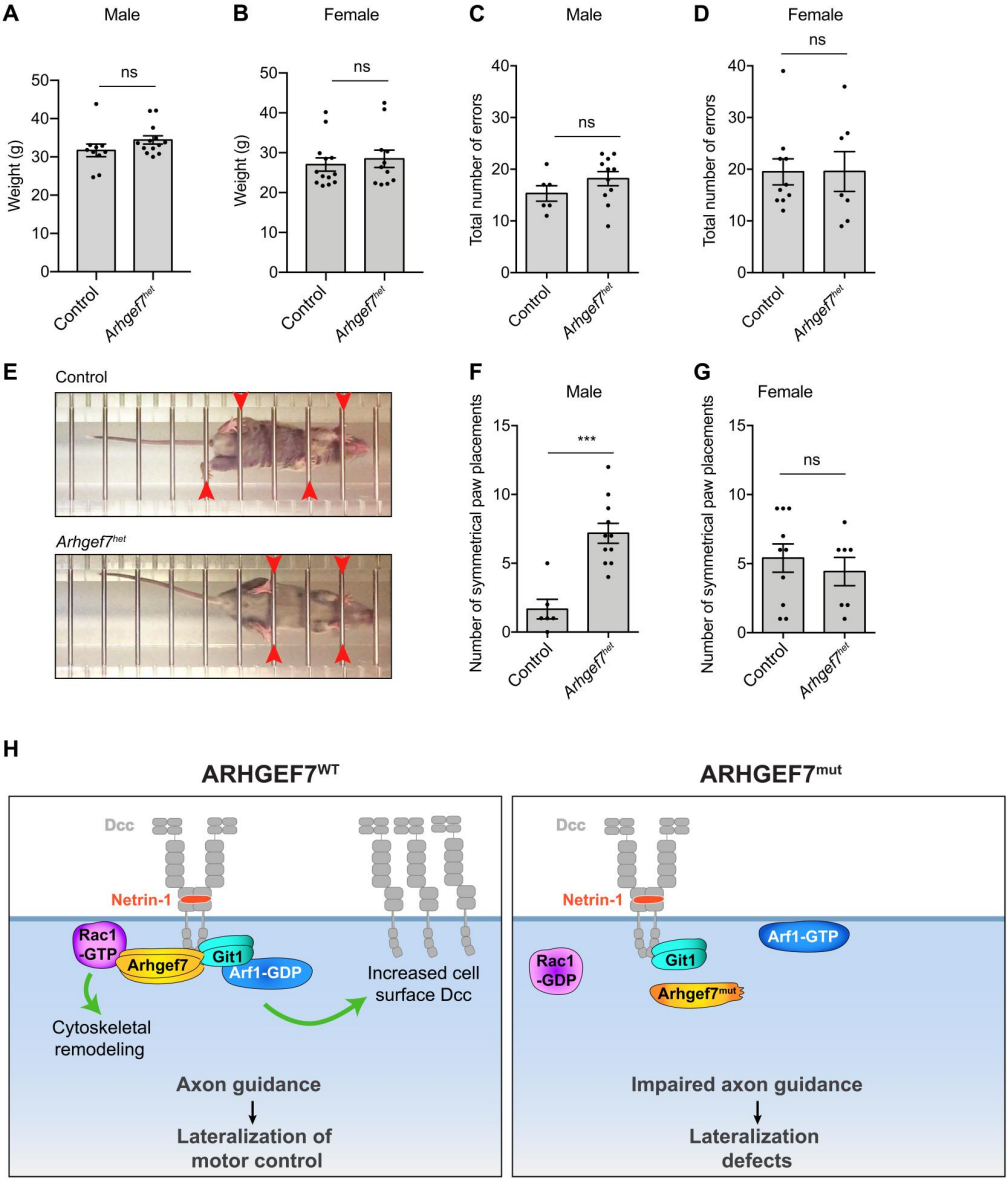
*Arhgef7^het^* mice have increased symmetrical paw placements. (**A**) Weight of control (*n* = 10) and *Arhgef7^het^* (*n* = 13) male mice. (**B**) Weight of control (*n* = 13) and *Arhgef7^het^* (*n* = 11) female mice. Mann-Whitney test. (**C** and **D**) The total number of errors made in the horizontal ladder rung test, for males and females. (**E**) Images of mice walking on the ladder rungs. Red arrows indicate the paw positions. Top: Image of a control mouse with typical asymmetrical (alternating) paw placements. Bottom: Image of an *Arhgef7^het^* mouse with symmetrical paw placements. (**F** and **G**) The number of symmetrical paw placements during the ladder rung test for males and females. (C, D, F, and G) Number of males: control (*n* = 6) and *Arhgef7^het^* (*n* = 11), and females: control (*n* = 10) and *Arhgef7^het^* (*n* = 7). Mann-Whitney, ***P < 0.001. (**H**) Arhgef7 forms a multifunctional effector complex required for Netrin-1–mediated axon guidance and lateralization of motor control. Arhgef7/Git1 directly bind to Dcc. Netrin-1 activates Rac1 and inactivates Arf1 in an Arhgef7/Git1-dependent manner. Arf1 inactivation increases cell surface Dcc. Arhgef7^mut^ does not bind to Dcc and Git1. When Arhgef7^mut^ is expressed, Netrin-1 fails to activate Rac1 and inactivate Arf1. Consequently, axon guidance is impaired resulting in lateralization defects.

Having found that *Arhgef7^het^* embryos have defects in their commissural axon trajectory, we performed locomotion studies on adult *Arhgef7^het^* mice to determine whether these defects correlated with a change in their motor behaviour. The weight of male and female *Arhgef7^het^* mice were not different from control mice, indicating that *Arhgef7^het^* mice have no gross defects in overall development (Fig. 8, A and B). We used the horizontal ladder rung walking test, where mice need precise limb trajectories and paw placements, to evaluate the precision of limb positioning and interlimb coordination in skilled walking (*52*, *53*). Mice were placed in a corridor with horizontal bars spaced 2 cm apart (“ladder rungs”) and allowed to walk on the bars. All errors in paw placement (partial placement, changes in placement, slips, misses, symmetry) were recorded. We found that the total number of errors made by male or female *Arhgef7^het^* mice were not different from control mice (Fig. 8, C and D), indicating that *Arhgef7^het^* mice have no gross defect in skilled walking. However, when we analyzed the number of symmetrical paw placements only (i.e., two paws placed simultaneously on the same rung), we found that male *Arhgef7^het^* mice, but not female *Arhgef7^het^* mice, had significantly more symmetrical paw placements compared to control mice (Fig. 8, E to G). This increase in symmetric movements in male *Arhgef7^het^* mice indicates that they have a defect in lateralized coordinated movements. This phenotype is consistent with *Arhgef7^het^* male, but not female, embryos having defects in their commissural axon trajectories (Fig. 7). Therefore, defects in the trajectory of commissural axons in male *Arhgef7^het^* mice are associated with with more synchronous movements during skilled walking, supporting *ARHGEF7* mutation as a cause for MM.

## DISCUSSION

We have identified a pathogenic variant in *ARHGEF7* that causes MM and delineated the mechanism via which *ARHGEF7* mutation causes MM. Arhgef7, together with its partner Git1, bind directly to the Netrin-1 receptor Dcc (Fig. 8H). We found that Arhgef7 GEF activity and Git1 GAP activity are required for Netrin-1–mediated attraction of spinal cord commissural axons. The Arhgef7/Git1 complex activates Rac1 and Cdc42, and inhibits Arf1 downstream of Netrin-1/Dcc. Arf1 is required for the Netrin-1–induced increase in cell surface Dcc. Unlike ARHGEF7^WT^, ARHGEF7^mut^ is unable to bind to Dcc and does not rescue axon attraction to Netrin-1; nor does it rescue the Netrin-1–induced increase in cell surface Dcc when *Arghef7* is knocked down, demonstrating that ARHGEF7^mut^ is a loss-of-function mutation (Fig. 8H). In vivo, *Arhgef7^het^* mice have defects in commissural axon trajectories in the embryonic spinal cord, consistent with impaired Netrin-1/Dcc signaling. Adult *Arhgef7^het^* mice have increased symmetrical paw placements during skilled walking, thus displaying a MM-like phenotype. In summary, *ARHGEF7* heterozygous disruption impairs Netrin-1/Dcc signaling in axon guidance, resulting in MM.

### Netrin-1/Dcc signaling regulates small GTPases to guide axons

Netrin-1/Dcc signaling activates the Rho GTPases Rac1 and Cdc42 (*21*–*24*) and inhibits RhoA (*54*). We found that the Netrin-1–induced activation of Rac1 and Cdc42 in commissural neurons requires Arhgef7. We also found that Netrin-1 inhibits Arf1 activity in commissural neurons, and that this also requires Arhgef7, presumably complexed with Git1, an ArfGAP. Until now, Arf1 has not been linked to Netrin-1/Dcc signaling or axon guidance.

Netrin-1 increases cell surface Dcc at the growth cone via a posttranslational mechanism (*44*). We show that Arf1, together with Arhgef7/Git1, is required for the Netrin-1–induced increase in cell surface Dcc. Given that Netrin-1 inhibits Arf1 activity, this suggests that a reduction in Arf1 activity leads to the increase in cell surface Dcc. Blocking this reduction in Arf1 activity by *Arhgef7* knockdown or Git1^R39A^ expression blocks the Netrin-1–induced increase in cell surface Dcc. However, *Arf1* knockdown also blocked the Netrin-1–induced increase in cell surface Dcc, suggesting that the cycling of Arf1 between the active GTP-bound and inactive GDP-bound state is important for its function (*49*), as has been shown in other systems where expression of a dominant-negative Arf1 that mimics the GDP-bound form and expression of a constitutively active Arf1 that mimics the GTP-bound form have the same phenotype as *Arf1* knockdown (*50*).

The effect of Arf1 on cell surface Dcc might be through inhibiting Dcc exocytosis or promoting Dcc endocytosis. Arf1 is predominantly localized at the Golgi apparatus where it regulates the assembly of coat complexes, organelle structure, and secretory trafficking (*55*). In addition to its localization at the Golgi apparatus, Arf1 has also been localized to early endosomes and recycling endosomes (*56*) and the plasma membrane (*57*–*59*). It has been suggested that Arf1 is required for endocytosis (*60*) and in *Drosophila*, it promotes clathrin-mediated endocytosis (*61*). Recent studies show that activation of Arf1 is required for the endocytosis of VE-cadherin upon endothelial junction destabilization (*62*). Git1 has also been implicated in the ligand-stimulated endocytosis of receptors, including several G protein–coupled receptors and the tyrosine kinase receptor epidermal growth factor receptor (*63*). Therefore, Arf1, regulated by Arhgef7/Git1, may promote Dcc endocytosis, and Netrin-1 inhibition of this process would lead to an increase in cell surface Dcc.

The recruitment of Dcc to the cell surface also occurs via RhoA inhibition (*54*). RhoA is activated by Arf1 in response to epidermal growth factor (*50*). Thus, the reduced Arf1 activity downstream of Netrin-1/Dcc signaling may also increase cell surface Dcc through an Arf1-dependent reduction in RhoA activity.

The interaction between Arhgef7/Git1 and Dcc is dynamic, increasing after 2 min of Netrin-1 stimulation and decreasing after 5 min of Netrin-1 stimulation. This may reflect the kinetics of Dcc localization to different cellular compartments (e.g., intracellular vesicles and cell surface) after Netrin-1 stimulation and the differential interaction of Arhgef7/Git1 with Dcc depending on the compartment Dcc is in.

Arhgef7 likely functions in concert with other GEFs to induce axon outgrowth and guidance downstream of Netrin-1/Dcc. Trio is a GEF that mediates Netrin-1 signaling in axon outgrowth and guidance through its ability to activate Rac1 (*23*). The phenotype of *Trio^−/−^* embryos is milder than that of *Dcc* and *Netrin-1* mutants (*23*), consistent with Trio being partially redundant with other GEFs, such as Arhgef7 (this manuscript) and Dock1 (*22*). In cortical neurons, Trio regulates the distribution of Dcc at the cell surface, specifically the ratio of cell surface Dcc at the growth cone relative to the axon (*64*), presumably via its effect on Rac1 activity, but it is not known whether it also regulates absolute levels of cell surface Dcc in the growth cone in response to Netrin-1, as does Arhgef7.

### *ARHGEF7* is a MM gene required for Netrin-1–mediated commissural axon guidance

We found that Arhgef7 forms a multifunctional effector complex required for Netrin-1/Dcc signaling in axon guidance and the lateralization of motor control in mice and that a pathogenic variant of *ARHGEF7* causes MM in humans. Though MM affect both males and females, males carrying *DCC* mutations exhibit MM more frequently than females (*5*, *65*), indicating that MM might be more penetrant in males than females. This is consistent with our results showing that male but not female *Arhgef7^het^* mice have a commissural axon guidance phenotype and display more symmetrical paw placements compared to control mice. However, the nine affected individuals in the *ARHGEF7^mut^* family are females. This contrasts with the male preponderance in MM individuals with *DCC* mutations (*5*, *65*). Notably, there is a sampling bias for members in the *ARHGEF7^mut^* family. We do not have the clinical and genetic data for all family members (particularly the males), and there are many more females in the third generation, which is the generation that was most examined. Furthermore, given the great variability in MM expression among affected members, with some family members being unaware of their MM before it being noted at their clinical examination (*15*), it is possible that other family members have MM but are unaware. Therefore, we cannot conclude whether there is a sex bias in the penetrance of *ARHGEF7^mut^* in this family.

The newly identified MM gene *ARHGEF7* functions in Netrin-1/DCC signaling, like the MM genes *DCC*, *NTN1*, and possibly *RAD51* (*5*, *12*, *13*, *16*). This highlights the importance of Netrin-1/DCC signaling in the formation of commissures and the lateralization of motor control. Loss of *Dcc* specifically in the spinal cord causes commissural axon misrouting and is sufficient to cause mice to have a hopping gait, a lateralization defect phenotype (*66*). Case studies of congenital MM suggests that the neuroanatomical defects are greatly variable and can be present in more than one neuronal structure involved in the motor pathway, including the corpus callosum (CC) and corticospinal tract (CST) (*4*). This is consistent with MM genes, such as *NTN1* and *DCC* being involved in the development of the CC and the CST, in addition to the guidance of spinal cord axons and formation of the spinal cord commissure (*4*). Given that *ARHGEF7* is required for Netrin-1/Dcc signaling in spinal cord commissural axon guidance, other Netrin-1/Dcc-dependent axon guidance events may be affected in the *Arhgef7^het^* mice, such as midline crossing of CST axons, and contribute to the MM-like phenotype (*67*, *68*).

Thus, a mutation in a GEF is sufficient to cause defects in axon guidance and MM, highlighting the importance of GEFs in human axon guidance. Our discovery of *ARHGEF7* as a MM gene illustrates the impact of using human genetics to identify molecular players in axon guidance.

## MATERIALS AND METHODS

### Human participants

This study was approved by the McGill University Heath Center Research Ethics Board (13-244-PED), and informed consent was obtained from each participant or legal guardian. Blood samples for DNA isolation were obtained from individuals II-2, II-11, III-1, III-4, III-8, III-10, III-13, III-14, and IV-6 (Fig. 1A).

### Animals

All animal work was performed in accordance with the Canadian Council on Animal Care Guidelines and approved by the IRCM (Montreal Clinical Research Institute) Animal Care Committee. Staged pregnant Sprague Dawley rats were obtained from Charles River (St. Constant, Canada). Embryos of both sexes (not determined) were randomly used for primary dissociated neuron culture. Mice were maintained in the IRCM specific pathogen–free animal facility. All mice were maintained on a C57BL/6 (the Jackson Laboratory, RRID:IMSR_JAX:000664) background. Mice harboring the *Arhgef7^tm1b(eucommWtsi)^* null allele and *Arhgef7^tm1c(eucommWtsi)^* conditional allele were provided by T. Omelchenko and K. V. Anderson (*51*). *Arhgef7*^−/f^ mice are referred to as *Arhgef7^het^* and *Arhgef7*^+/f^ are the control mice. E0 was defined as midnight of the night before a plug was found. Embryos of both sexes were used for analysis of spinal cord commissural axon tracts, and their sex was determined by genotyping (*69*).

### Primary commissural neuron culture

Dissociated commissural neuron cultures were prepared as previously described (*34*, *70*). Briefly, tissue culture plates or acid-washed and sterilized glass coverslips were coated with poly-L-lysine (PLL) (100 μg/ml for 2 hours). The dorsal fifth of E13 rat neural tubes were microdissected and quickly washed once in cold Ca^2+^/Mg^2+^-free Hanks’ balanced salt solution (HBSS). The tissue fragments were trypsinized with 0.15% trypsin in Ca^2+^/Mg^2+^-free HBSS for 7 min at 37°C. Deoxyribonuclease (Worthington, LS002139) was added briefly. The tissue fragments were then washed in warm Ca^2+^/Mg^2+^-free HBSS and triturated in Ca^2+^/Mg^2+^-free HBSS to yield a suspension of single cells. Cells were plated in Neurobasal media supplemented with 10% heat-inactivated fetal bovine serum (FBS) and 2 mM GlutaMAX (Life Technologies, 35050-061). After ~21 hours, the medium was changed to Neurobasal supplemented with 2% B27 (Life Technologies, 17504-044) and 2 mM GlutaMAX. Commissural neurons were used for experiments after 2 days of culture in vitro. For Dunn chamber experiments, electroporated commissural neurons were plated at 180,000 to 240,000 cells per well in six-well plates on acid-washed PLL-coated 18-mm square #3D coverslips (Assistent, Germany). For immunostaining, commissural neurons were plated at 35,000 cells per well in 24-well plates on acid-washed PLL-coated 12-mm round #1D coverslips. For biochemical experiments, commissural neurons were plated at 1.5 × 10^6^ to 2 × 10^6^ cells per well in PLL-coated six-well plates.

### Cell lines

Cos7 cells were maintained in Dulbecco’s modified Eagle’s medium +10% FBS + penicillin/streptomycin (Invitrogen) in a 5% CO_2_ humidified incubator. The cell lines have not been authenticated.

### Screening of family for variants in known MM genes

DNA was isolated from whole blood samples obtained from the individuals using the Qiagen Puregene Blood Core C Kit (MD, USA). We screened one affected family member for mutations in the four genes associated with congenital MM: *DCC*, *RAD51*, *NTN1*, and *DNAL4*. Primer pairs were designed with Primer 3 Plus for amplifying exonic regions and exon/intron boundaries. Multiplex polymerase chain reactions (PCRs) were performed, and libraries prepared with the Illumina Nextera XT DNA Sample Preparation Kit (Illumina, Vancouver, Canada). Libraries were then paired end–sequenced in reactions of 150–base pair (bp) reads on the MiSeq using 300-cycle reagent kits (Illumina, Vancouver, Canada), and bioinformatic analyses were performed. Variants identified by next-generation sequencing were also validated by PCR amplification and Sanger sequencing (Genome Quebec, Montreal, QC, Canada). Exons and flanking regions were amplified by PCR.

### Exome sequencing and bioinformatic analysis

Blood genomic DNA from family members was captured with the Agilent SureSelect Human All Exon Capture V4 Kit and sequenced (two paired-end 100-bp reads, three exomes per lane) with Illumina HiSeq 2000 at the McGill University Genome Quebec Innovation Center (Montreal, Canada). Sequence processing, alignment (with a Burrows-Wheeler algorithm), and variant calling were done according to the Broad Institute Genome Analysis Toolkit (GATK v.4) best practices, and variant annotation was done with ANNOVAR (*71*). The average exome coverage of the target bases was 111 to 143×, and 95% of the target bases were covered by at least 20 reads. Only the variants whose positions were covered ≥8 and supported by at least three variant reads constituting at least 20% of the total reads for each called position were retained. To identify potentially pathogenic variants, we filtered out (i) synonymous variants or intronic variants other than those affecting the consensus splice sites, (ii) variants seen in more than 2% of our inhouse exomes (*n* = 1000) from unrelated projects, and (iii) variants with a minor allele frequency greater than 0.5% in gnomAD. We then considered only variants that were shared by all seven MM individuals who were affected or obligate carriers. The c.1751_1752del, p.Asn584Thrfs*90, in *ARHGEF7* (NM_001113511.1) was the only variant that was absent in gnomAD and shared by the seven affected individuals.

### Reagents

Netrin-1 was purchased from R&D Systems (1109-N1-025, Minneapolis, MN) and BSA from MultiCell (500-0206). They were used at 100 ng/ml. Unless specified, Netrin-1 stimulations were performed for 5 min. EHT1864 was purchased from Selleckchem (S7482, Batch: 5748201). TRITC-phalloidin was purchased from Sigma-Aldrich (P1951), and Alexa Fluor 488-phalloidin was purchased from Thermo Fisher Scientific (A12379).

### Plasmids

pcDNA3-Rat Git1^WT^-FLAG Cterm was a gift from R. T. Premont and A. Claing (*72*). pcDNA3-Rat Git1^R39A^-FLAG Cterm was derived from pcDNA3-Rat Git1^WT^-FLAG Cterm using In-Fusion (Clontech 639648) to change Rat Git1 bp 115 to 117 from CGG (arginine) to GCT (alanine). Rat Git1^WT^-FLAG Cterm and Rat Git1^R39A^-FLAG Cterm were also subcloned into pCAGGS for commissural neuronal expression. pCMV5-Human ARHGEF7^WT^-FLAG (coding sequence corresponds to transcript variant 5 that encodes ARHGEF7 isoform a), pCMV5-Human ARHGEF7 ΔSH3-DH-FLAG, pCMV5-Human ARHGEF7 ΔKER-LZ-FLAG, pCMV5-Human ARHGEF7 ΔKER-FLAG, and pCMV5-Human ARHGEF7 ΔLZ-FLAG were a gift from L. Attisano (*35*). pCMV5-Human ARHGEF7^mut^-FLAG was made by GenScript USA Inc. using site-directed mutagenesis to delete CC at positions 1217 to 1218 of human *ARHGEF7* from pCMV5-Human ARHGEF7^WT^-FLAG. We used In-Fusion to transfer ARHGEF7^WT^-FLAG and ARHGEF7^mut^-FLAG from pCMV5 into pCAGGS for robust expression in commissural neurons and into pcDNA3.1, that has a T7 promoter for in vitro transcription. pCAGGS-human ARHGEF7^GD^-FLAG, the GEF-dead variant (L238R/L239S) (coding sequence corresponds to transcript variant 5) (*29*), was derived from pCAGGS-ARHGEF7^WT^-FLAG using In-Fusion to change ARHGEF7 bp 712 to 714 from CTG (leucine) to CGG (arginine) and bp 715 to 717 from CTC (leucine) to TCC (serine). pcDNA3.1-Rat Dcc-3xHA encoding the full-length coding sequence of rat Dcc (bp 1 to 4338, or amino acids 1 to 1446), with 3xHA at the 3′ end, was obtained from M. Tessier-Lavigne. pcDNA3.1-Rat Dcc Δcyto-3xHA was constructed by subcloning bp 1 to 3375 of the rat Dcc coding sequence (which corresponds to amino acids 1 to 1125, encompassing the extracellular domain, the transmembrane domain, and three amino acids of the cytoplasmic tail), with 3xHA at the 3′ end, into the pcDNA3.1 vector using Bam HI–Not I restriction sites. pCAGGS-GFP was constructed in our laboratory. pGEX-4 T1 GST was obtained from Cytiva Life Sciences (28-9545-49). pGEX GST-Tev-human DCC cytoplasmic tail long (1126 to 1447 amino acids), GST-Tev-human DCC cytoplasmic tail medium (1163 to 1447 amino acids, ∆P1), GST-Tev-human DCC cytoplasmic tail short (1274 to 1421 amino acids, ∆P1, and ∆P3), GST-Tev-human DCC P1 domain (1126 to 1176 amino acids) and GST-Tev-Frazzled (*Drosophila*) (1252 to 1526 amino acids) were derived from pGEX-Tev using Nde I/Bam HI restriction sites (*73*). pGEX-4 T2 GST-Rat-Dcc (Dcc cytoplasmic domain only) was a gift from N. Lamarche-Vane (*74*). pGEX-4 T GST-PAK (CRIB) and pGEX-GST-GGA3 were a gift from A. Claing (*57*, *75*). pCAGGS-human ARF1^WT^-HA and pCAGGS-human ARF1^T31N^-HA were derived from pcDNA3-human ARF1^WT^-HA Cterm [a gift from A. Claing (*50*)] using In-Fusion to change ARF1 bp 91 to 93 from ACC (threonine) to AAC (asparagine).

### Electroporation of commissural neurons

Commissural neurons were electroporated with the Amaxa 96-well Shuttle using the P3 Primary Cell 96-well Nucleofector Kit (Lonza, Switzerland). For each electroporation in one well (20 μl) of a 96-well Nucleofector Plate, 0.75 × 10^6^ to 1 × 10^6^ commissural neurons were electroporated with 0.5 μg of plasmid DNA or 1 μM siRNA. The electroporation was performed with the program 96-CP-100 according to the manufacturer’s instructions.

### shRNA generation and validation

shRNA with a microRNA stem (shRNAmir) for knockdown of rat *Arhgef7* was generated by ligating oligonucleotides encoding the target sequence into the pcDNA6.2-GW/EmGFP-miR vector. The EmGFP-miR cassette was then subcloned into the pCAGGS vector. The efficiency and specificity of the shRNA was evaluated in vitro in neurons (fig. S1, A, B, E, and F). The target sequence for rat *Arhgef7* was identified using BLOCK-iT RNAi Designer (Thermo Fisher Scientific): 5′-GCTGGTGAGGAAGGTTCTAAA-3′.

### Small interfering RNA

siRNAs were designed using the Custom Dicer-Substrate siRNA (DsiRNA) system (IDT). siRNA oligonucleotides were annealed by incubation at 94°C for 2 min and cooling down at room temperature, then aliquoted and stored at −20°C.

Scrambled siRNA.

5′-rUrCrArCrArArGrGrGrArGrArGrArArArGrArGrArGrGrArArGrGrA-3′.

5′-rCrUrUrCrCrUrCrUrCrUrUrUrCrUrCrUrCrCrCrUrUrGrUGA-3′.

*Arf1* siRNA a.

5′- rArGrArGrArArUrCrArArCrUrCrArCrUrGrUrCrArGrUrArCrCr-3′.

5′-rGrGrUrArCrUrGrArCrArGrUrGrArGrUrUrGrArUrUrCrUrCrUrUrU-3′.

*Arf1* siRNA b.

5′-rArArCrGrUrGrGrArGrArCrUrGrUrUrGrArArUrArCrArArGrA-3′.

5′-rUrCrUrUrGrUrArUrUrCrArArArGrUrCrUrCrCrCrGrUrUrGrA-3′.

*Arf1* siRNA a and b were validated in commissural neurons (fig. S8, A and B).

Git1 siRNA a.

5′-rArGrArArUrGrGrGrCrArUrUrArCrArUrCrAUrArCrCrArCrA-3′.

5′-rUrGrUrGrGrUrArUGrArUrGrUrArArUrGrCrCCrArUrUrCrUrUrG-3′.

Git1 siRNA b.

5′-rArArGrArUrGrArUrGrCrCrArUrCrUrArUrUrCrArGrUrArCrA-3′.

5′-rUrGrUrArCrUrGrArArUrArGrArUrGrGrCrArUrCrArUrCrUrUrCrC-3′.

Git1 siRNA c.

5′-rCrArGrUrGrUrGrGrCrUrArGrCrUrArCrCrCrArGrArArUrCrA-3′.

5′-rUrGrArUrUrCrUrGrGrGrUrArGrCrUrArGrCrCrACrArCrUrGrCrA-3′.

### Antibodies

Antibodies against the following targets were used: Robo3 (R&D Systems, AF3076, 1:200 for immunofluorescence (IF)), Rac1 (Millipore 05-389, clone 23A8; 1:2000 for WB), HA (Sigma-Aldrich, H3663; clone 12CA5, 1:2000 for WB, 1:1000 for IF), Flag (Sigma-Aldrich, F3165; 1:2000 for WB, 1:1000 for IF, 2 mg for IP), ARF1 (Proteintech, 10790-1-AP, 1:1000 for WB), ARF6 (Santa Cruz Biotechnology, sc-7971, clone 3A1, 1:500 for WB), GIT1 (Novus Bio, NBP2-22423, clone S39B-8, 1:1000 for WB, 1:100 for IF), Dcc extracellular domain (Dcc_ex_) (Calbiochem, OP45, clone AF5, 1:50 for IF), DCC epitope mapping near the C terminus of DCC of human origin (Santa Cruz Biotechnology, sc-6535, clone A-20, 1:500 for WB, 1:200 for IF, 1:50 for PLA), β-Pix/ARHGEF7 (MilliporeSigma, 07-1450-I, 1:1000 for WB, 1:200 for IF, 1:50 for PLA, 2 mg for IP), GFP (Molecular Probes, A11122, 1:2000 for WB and IF), pan-actin [Cell Signaling (NEB), sc-138, 1:1000 for WB], GST (Santa Cruz Biotechnology, sc-138, clone B14, 1:1000 for WB), Cdc42 (Santa Cruz Biotechnology, sc-87, 1:1000 for WB), and Isl1/2 (DSHB 39.4D5-b, 1:100 for IF).

### Western blotting

Cells were lysed with LMB [25 mM Hepes (pH 7.5), 150 mM NaCl, 1% NP-40, 10 mM MgCl_2_, 1 mM EDTA, and 10% glycerol] with protease inhibitors (Roche, 11873580001) and boiled in SDS sample buffer for 5 min. Protein samples were separated by SDS–polyacrylamide gel electrophoresis (SDS-PAGE) and transferred to polyvinylidene difluoride membrane. The membranes were blocked with 5% skim milk in TBST [0.01 M tris-HCl (pH 7.5), 150 mM NaCl, and 0.1% Tween 20], followed by primary antibody incubation in 5% BSA (Wisent, 800-095-EG) in TBST. Secondary antibodies were conjugated to horseradish peroxidase and Western blots were visualized with chemiluminescence.

### Immunostaining

For immunostaining of dissociated neuron cultures, neurons were gently fixed with 4% paraformaldehyde (PFA) at 37°C for 15 or 9 min for nonpermeabilized staining. For immunostaining of mouse embryos, embryos were collected at E7.5, E10.5, or E11.5 and fixed for 2 hours in 4% PFA on ice. After three washes in phosphate-buffered saline (PBS), the embryos were placed in a 30% sucrose solution overnight to cryoprotect the tissues and then embedded in OCT and frozen. Sections of E7.5 embryos were cut at a thickness of 8 μm. Sections of E10.5 and E11.5 embryos were cut at a thickness of 10 to 16 μm at the forelimb level using a cryostat. Dissociated neurons or tissue sections were blocked for 1 hour with 10% donkey serum with 0.1% Triton X-100 in PBS at room temperature. Antibodies were then incubated at 4°C in 1% donkey serum and 0.1% Triton X-100 in PBS overnight. For nonpermeabilized staining, no Triton was used. After three washes with PBS, the secondary antibodies were incubated in the same solution as the primary antibodies. Nuclei were stained with 4′,6-diamidino-2-phenylindole (Sigma-Aldrich, D95964), and samples were mounted in Mowiol 4-88 (Sigma-Aldrich, 81381). Images of fluorescence immunostaining of commissural neuron cultures were obtained with a Zeiss LSM 700 confocal microscope with either a 40× or a 63× objective, and images of fluorescently stained spinal cord sections were obtained with a Leica SP8 or Zeiss LSM 700 confocal microscope with a 20× objective.

### Proximity ligation assay

Dissociated commissural neurons were stimulated with BSA or Netrin-1 for 2 or 5 min and fixed with 4% PFA in PBS. The samples were blocked with 10% BSA [immunoglobulin G (IgG) free] and 0.1% Triton X-100 in PBS (pH 7.4) for 1 hour at room temperature and then incubated with antibodies against DCC (1:50) (Santa Cruz Biotechnology sc-6535) and ARHGEF7 (1:50) (MilliporeSigma, 07-1450-I) or GIT1 (1:50) (Novus Bio NBP2-22423), diluted in PBS with 1% BSA (IgG free) and 0.1% Triton X-100, overnight at 4°C. The proximity ligation reaction was performed with the Duolink in situ PLA kit (Sigma-Aldrich) according to the manufacturer’s instructions.

### GTPase activation assay

Cos7 cells were transfected with empty vector, ARHGEF7^WT^-FLAG or ARHGEF7^mut^-FLAG and were cultured for 2 days in vitro. Alternatively, commissural neurons were electroporated with scrambled and *Arhgef7* shRNAmir immediately after dissociation and cultured for 2 days in vitro and then stimulated with BSA (Sigma-Aldrich, A4161) or Netrin-1 for 5 min. Briefly, cells were washed with cold tris-buffered saline (TBS) and lysed in 700 μl of ice-cold lysis buffer E [50 mM tris-HCl (pH 7.4), 1% Nonidet P-40, 137 mM NaCl, 10% glycerol, 5 mM MgCl_2_, 20 mM sodium fluoride, and 1.0 mM Na_3_VO_4_ with protease and phosphatase inhibitors]. Samples were vortexed for 10 s and centrifuged for 10 min at 10,000*g*. The protein lysates were then incubated with 5 μg of GST-PAK (purified p21-binding domain of PAK expressed as a GST fusion protein) or GST-GGA3 conjugated to glutathione-sepharose beads for 45 min at 4°C to pull down active GTP-bound GTPases. Beads were washed and proteins were eluted in 30 μl of SDS sample buffer by heating at 65°C for 15 min. The eluted proteins were separated by SDS-PAGE. Detection of Rac1-GTP, Cdc42-GTP, or ARF1-GTP were performed by immunoblot analysis using specific anti-Rac1, anti-Cdc42, or anti-ARF1 antibodies. The amount of active GTPases for each sample was normalized to the total amount of the corresponding GTPase protein the cell lysate. A similar methodology was used for the detection of activated Rac1 in conditions where cells were stimulated with Netrin-1 for 5 min and treated with the Rac1 inhibitor EHT1864 (Selleckchem, S7482; batch: 5748201). For those experiments, commissural neurons were first treated with vehicle (H_2_O) or EHT1864 (10 or 20 μM) for 2 hours before being stimulated.

### Coimmunoprecipitation

Cos7 cells were transfected with the indicated expression vectors using Lipofectamine 3000 (Life Technologies L3000-015). Forty-eight hours after transfection, cells were lysed with LMB lysis buffer (see above). Protein lysate (1 mg) in 700 μl of LMB buffer with protease and phosphatase inhibitors was incubated with 2 μg of the indicated antibodies for 2 hours to overnight at 4°C. Protein A/G-agarose beads (Santa Cruz Biotechnology, sc-2003) were added for 1 hour to capture the immunoprecipitated protein complex. For commissural neurons, cells were lysed with SLB buffer [10 mM tris (pH 8), 150 mM NaCl, and 0.5% Igepal]. The lysate was preincubated with protein A/G-agarose bead for 30 min to reduce unspecific binding, and the supernatant was collected. Protein lysate (400 μg) in 500 μl of SLB buffer with protease and phosphatase inhibitors was incubated with 1.15 μg of the indicated antibodies and protein A/G-agarose beads for 3 hours. The beads were washed three times with lysis buffer and proteins binding to the beads were eluted by adding SDS sample buffer and heating at 95°C for 5 min. The immunoprecipitated proteins were analyzed by SDS-PAGE and WB.

### In vitro pull-down assays

Pull-down assays were performed using immobilized, bacterially produced GST fusion proteins (*73*, *74*). GST, GST-DCC/Frazzled cytoplasmic domain constructs, and GST-Dcc were produced in BL21(DE3)pLysS (Novagen). The bacteria were lysed in 100 mM tris (pH 8.0), 10% sucrose, and 1.5 mM EDTA with lysozyme (100 μg/ml). The proteins were then incubated in extraction buffer [10 mM tris (pH 8.0), 0.16 mM EDTA, 1% sucrose, 50 mM NaCl, 0.5 mM DTT, and 0.2% N-Lauroylsarcosine (Sigma-Aldrich, L9150)], sonicated three times for 30 s each and then centrifuged. The supernatants were then incubated with 1% Triton, 1.25 mM MgCl_2,_ and 1 mM CaCl_2_ and were centrifuged once again. The supernatants were incubated with glutathione-agarose beads (Pierce 16100) and the protein-bound GST beads washed in PBS with 1% Triton, then in PBS containing 0.02% imidazole. The purity and yield of the GST-fusion proteins bound to the beads was confirmed by SDS-PAGE and Coomassie staining.

To test direct protein-protein interaction, Arhgef7 and Git1 proteins were synthesized using the in vitro transcription/translation (IVT) kit TnT Coupled Reticulocyte Lysate Systems (T7 Promoter) (Promega L4610) from pcDNA3-Human ARHGEF7^WT-^FLAG, pcDNA3-Human ARHGEF7^mut^-FLAG, and pcDNA3-Rat Git1^WT^-FLAG plasmids, respectively. Yield and purity was confirmed by SDS-PAGE and Western blot. Depending on the yield, between 25 and 40 μl of IVT proteins were combined with 3 μg of beads coupled to GST or the GST fusion proteins and incubated for 2 hours at 4°C in 0.1% IPH buffer [50 mM tris (pH 8.0), 50 to 150 mM NaCl, 5 mM EDTA, 0.01% BSA, and 0.1% NP40, with protease and phosphatase inhibitors]. The beads were then washed three times in 0.1% IPH buffer and boiled in 2× Laemmli buffer at 95°C for 5 min. The isolated proteins were analyzed by SDS-PAGE and WB.

### Dunn chamber assay and analysis

To quantify the growth cone turning of commissural neurons in response to gradients, we performed the Dunn chamber axon guidance assay as described previously (*34*). Electroporated commissural neurons were grown on PLL-coated square #3D coverslips as described above. The coverslips were then assembled into Dunn chambers. Gradients were generated in the Dunn chamber with Netrin-1 (100 ng/ml), or BSA (the vehicle for Netrin-1) in the outer well. After Dunn chamber assembly, time-lapse phase-contrast images were acquired for 2 hours at 37°C with a 10× fluotar on a Leica DMIRE2 inverted microscope (Leica, Germany) equipped with a MS-2000 XYZ automated stage (ASI, Eugene, OR). Images were acquired with an Orca ER CCD camera (Hamamatsu) using Volocity (Improvision, Waltham, MA). The angle turned was defined as the angle between the original direction of the axon and a straight line connecting the base of the growth cone from the first to the last time point of the assay period.

### DiI axon tracing

Spinal cords were dissected from E13.5 mouse embryos and fixed for 2 hours at room temperature in 4% PFA in PBS. After fixation, small 1,1′-dioctadecyl-3,3,3′,3′- tetramethylindocarbocyanine perchlorate (DiI, D282 Thermo Fisher Scientific, D282) crystals were inserted in the medial neural tube dorsal of the motor column to label ~5 to 9 individual cohorts per embryo at multiple levels along the anteroposterior axis (*76*). The DiI was allowed to diffuse for 2 to 3 days at room temperature. After diffusion of the dye, the spinal cords were mounted as open-book and imaged on a Leica SP8 confocal microscope with a 40× objective. Individual axon trajectories were analyzed using ImageJ (National Institutes of Health, USA) and the SNT plugin (*77*). Axons deviating from a straight trajectory while crossing the floor plate (either transiently traveling along the ipsilateral tract before entering the floor plate, making strong turns within the floor plate, and/or crossing the floor plate diagonally) were counted as aberrant crossing. The analysis was performed blind to the genotype and sex of the embryos.

### Locomotion testing

Locomotion studies were performed on adult male and female mice (9 to 20 weeks old). All the locomotion tests were performed blind to the genotype. The horizontal ladder rung test apparatus was used to evaluate the precision and coordination of limb positioning during skilled walking (*52*). The ladder test apparatus consists of a corridor 34 cm long and 5 cm wide with bars spaced 2 cm apart at the bottom, forming the horizontal ladder. In addition to the 34 cm corridor, there is a 12-cm-long turning zone with bars spaced 1 cm apart at each end of the ladder. The mice were made to cross the corridor and the position of the forelimbs and hindlimbs were recorded by video (iPhone 11 Pro or iPad Air). One crossing from one end of the ladder to the other end was considered one passage and five passages were analyzed for each mouse. The videos were then examined frame-by-frame, and the number of errors were recorded. The following errors were recorded [adapted from (*52*, *53*)]: Partial Placement: The limb was placed on the rung with either the wrists/digits of the forelimb or toes/heel of the hindlimb. Correction: The limb aimed for a rung, but it was placed on another one without touching the one initially aimed at. Replacement: The limb was placed on a rung, but before it could start weight bearing, it was lifted quickly and placed on another rung. Symmetry: Two limbs at the same time on the same rung. Slip: The limb paw was initially placed on a rung but slipped. Miss: The paw missed the rung.

### Image analysis

Quantification of the intensity of immunofluorescence images was performed with ImageJ (National Institutes of Health, USA). The average intensity of the region of interest was always background-corrected by subtracting the average intensity of a region of the coverslip.

For the in vivo axon guidance phenotype measurements, we calculated the ratio of the area occupied by Robo3^+^ commissural axons in the ventral third of the spinal cord relative to the total area of the ventral third of the spinal cord. We traced the edges of the commissural axonal tract on Robo3-immunostained E11.5 embryo cross sections using Fiji software. The width of each commissural axon tract was measured at a position one-sixth of the height of the spinal cord, from the floor plate. The analysis was performed blind to the genotype and sex of the embryos.

### Quantification and statistical analysis

Quantification of the digital images obtained by Western blot analysis was performed using ImageJ. Student’s *t* test or the Mann-Whitney test was used when there were only two groups in the dataset. To compare multiple groups in a dataset, statistical analyses were performed using a one-way or two-way analysis of variance followed by an appropriate multiple comparison test, using GraphPad Prism 7 (La Jolla, CA). All error bars represent SEM, and asterisks (*) indicate significance as follows: *P < 0.05, **P < 0.01, ***P < 0.001, ns, not significant (*P* > 0.05). The statistical analysis used in each experiment and the definition of *n* are stated in the figure legends.

## Notes

View/request a protocol for this paper from *Bio-protocol*.
