# Supplementary material for: Genetics of mirror movements identifies a multifunctional complex required for Netrin-1 guidance and lateralization of motor control
Source: Sci Adv. 2023 May 12;9(19):eadd5501. doi: 10.1126/sciadv.add5501 (PMC10181192; doi:10.1126/sciadv.add5501)
Supplement: Supplementary file 1 — Figs. S1 to S9 [file sciadv.add5501_sm.pdf]

Science Advances
AAAS

Supplementary Materials for

Genetics of mirror movements identifies a multifunctional complex required for Netrin-1 guidance and lateralization of motor control

Sabrina Schlienger *et al.*

Corresponding author: Frederic Charron, frederic.charron@ircm.qc.ca; Myriam Srour, myriam.srour@mcgill.ca

Sci. Adv. **9**, eadd5501 (2023)
DOI: 10.1126/sciadv.add5501

This PDF file includes:

Figs. S1 to S9

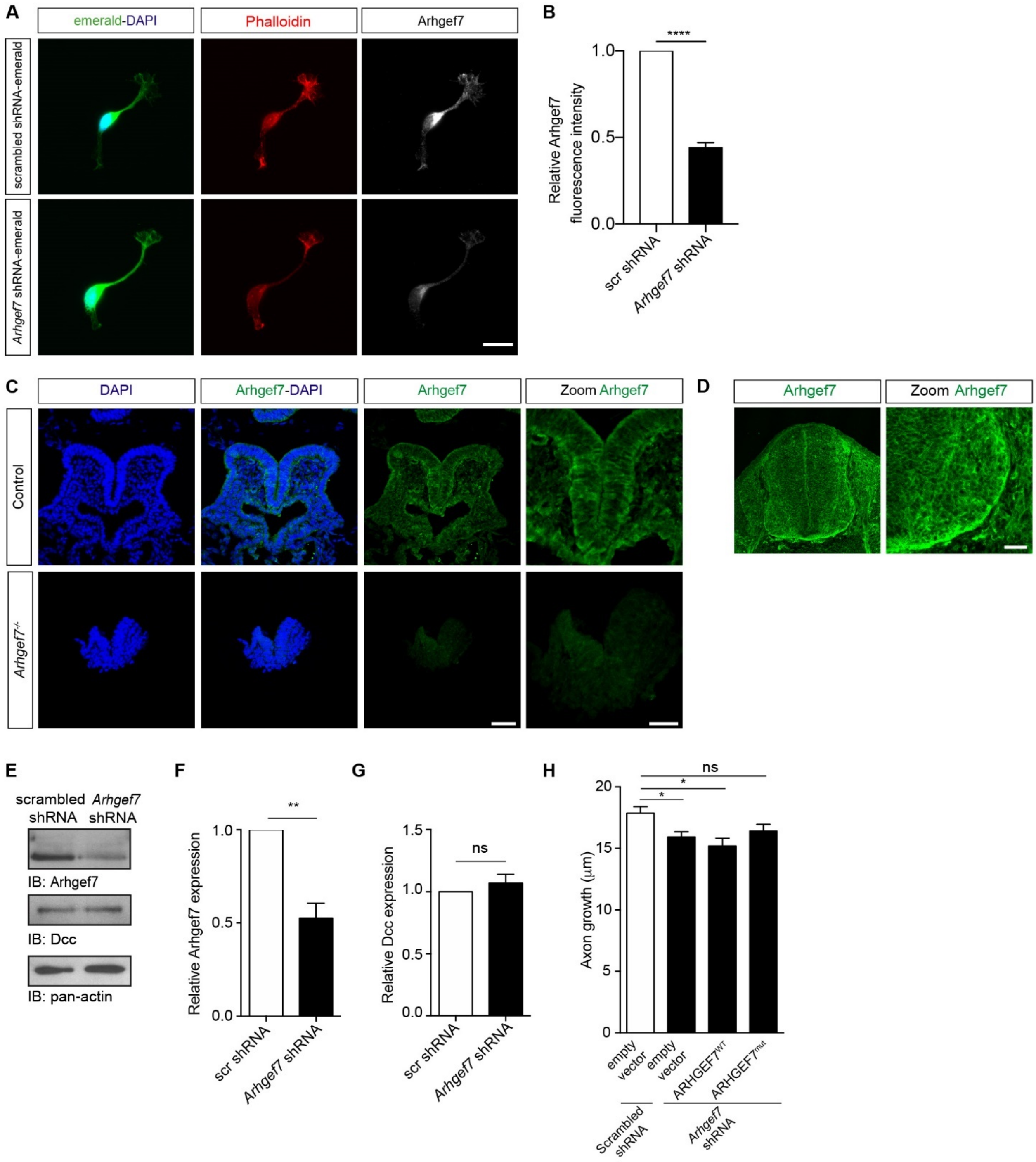

Fig. S1. Validation of Arhgef7 antibody and *Arhgef7* shRNAmir knockdown.

Related to Fig. 2.

(A) Commissural neurons were transfected with scrambled shRNA or *Arhgef7* shRNA and fixed and immunostained for Arhgef7 and GFP. F-actin was detected with phalloidin. Scale bar: 20 µm. **(B)** The mean (±SEM) intensity of Arhgef7 immunofluorescence signal. n = 4 experiments, 15 growth cones per condition, per experiment. Paired t test, ****p<0.0001. **(C)** E7.5 control or *Arhgef7*$^{-/-}$ embryos were immunostained for Arhgef7 and DAPI. Scale bar: 100 µm, zoom 50

µm. **(D)** Mouse E11.5 neural tube cross-sections were immunostained for Arhgef7. Scale bar: zoom 25 µm. **(E)** Commissural neurons were transfected with scrambled shRNA or *Arhgef7* shRNA. After 48 h, *Arhgef7* shRNA efficiently knocked down expression of Arhgef7 as assessed by Western blotting. **(F, G)** The relative amount (mean ± SEM) of Arhgef7 and Dcc protein normalized to the amount of pan-actin in the cell lysate. n=4, paired t test, $^{**}p<0.01$. **(H)** Commissural neurons were transfected with scrambled shRNA or *Arghef7* shRNA, together with empty vector, $ARHGEF7^{WT}$ or $ARHGEF7^{mut}$ expression vectors as indicated and then exposed to a gradient of Netrin-1 (0.1 µg/ml in the outer well) in the Dunn chamber. The axon growth (mean ± SEM) over 2 h was measured. n=132, 152, 63, 115, respectively. One-way ANOVA, Tukey's multiple comparison post-test, $^{*}p<0.05$.

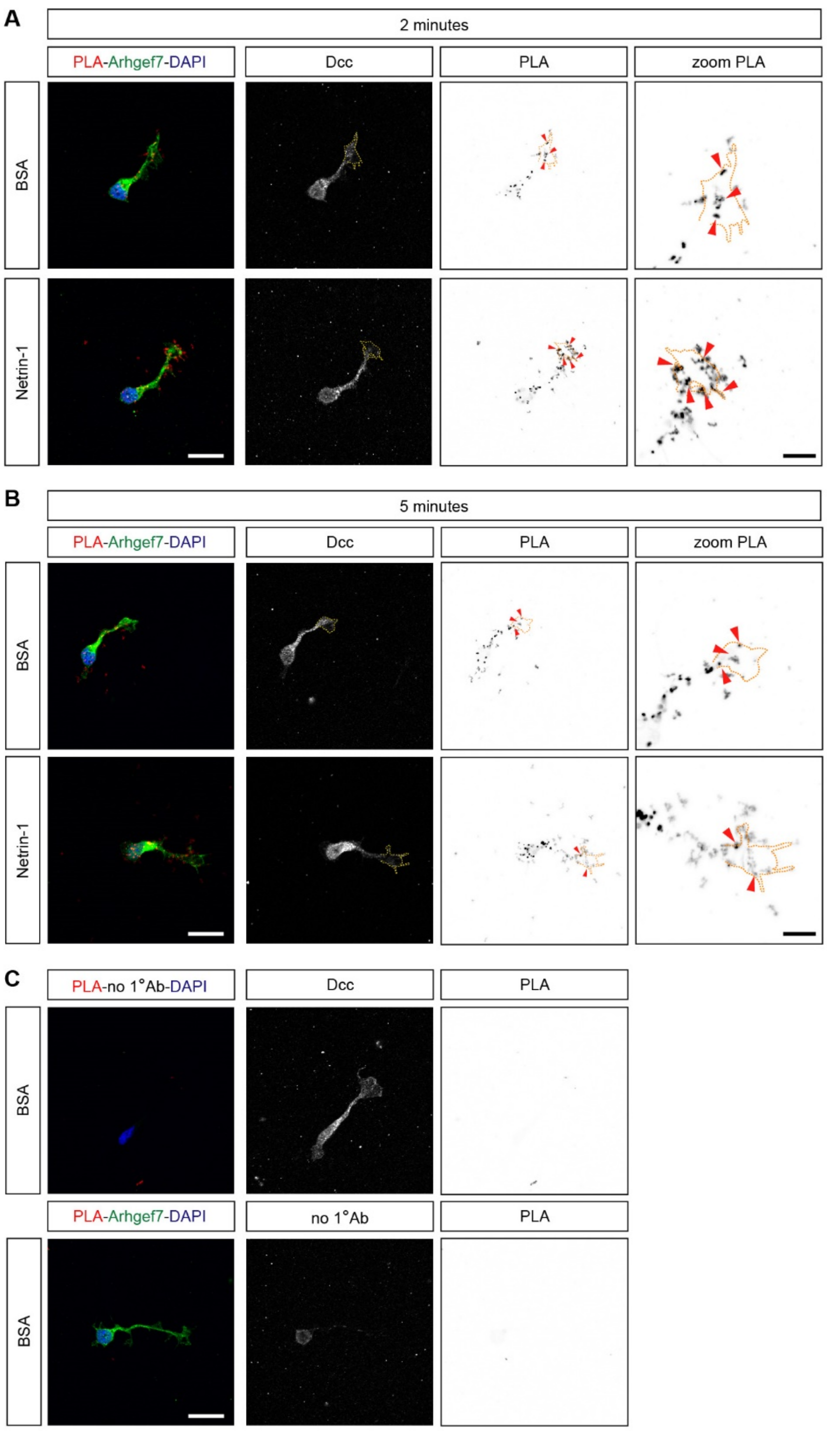

Fig. S2. Arhgef7 interacts with Dcc in growth cones, and this interaction is modulated by Netrin-1.

Related to Fig. 3.

(A, B) Dissociated commissural neurons were treated with 0.1 µg/ml BSA or Netrin-1 for 2 and 5 min respectively, then fixed with 4% PFA. The PLA assay was performed for Dcc and Arhgef7. After the PLA reaction, Dcc and Arhgef7 were counter-stained for visualization. Scale

bar: 20 µm (left), 7 µm (zoom). **(C)** Omission of one of the two primary antibodies to control for potential non-specific binding of the secondary antibodies and the quality of PLA probes. Data are representative of four independent experiments.

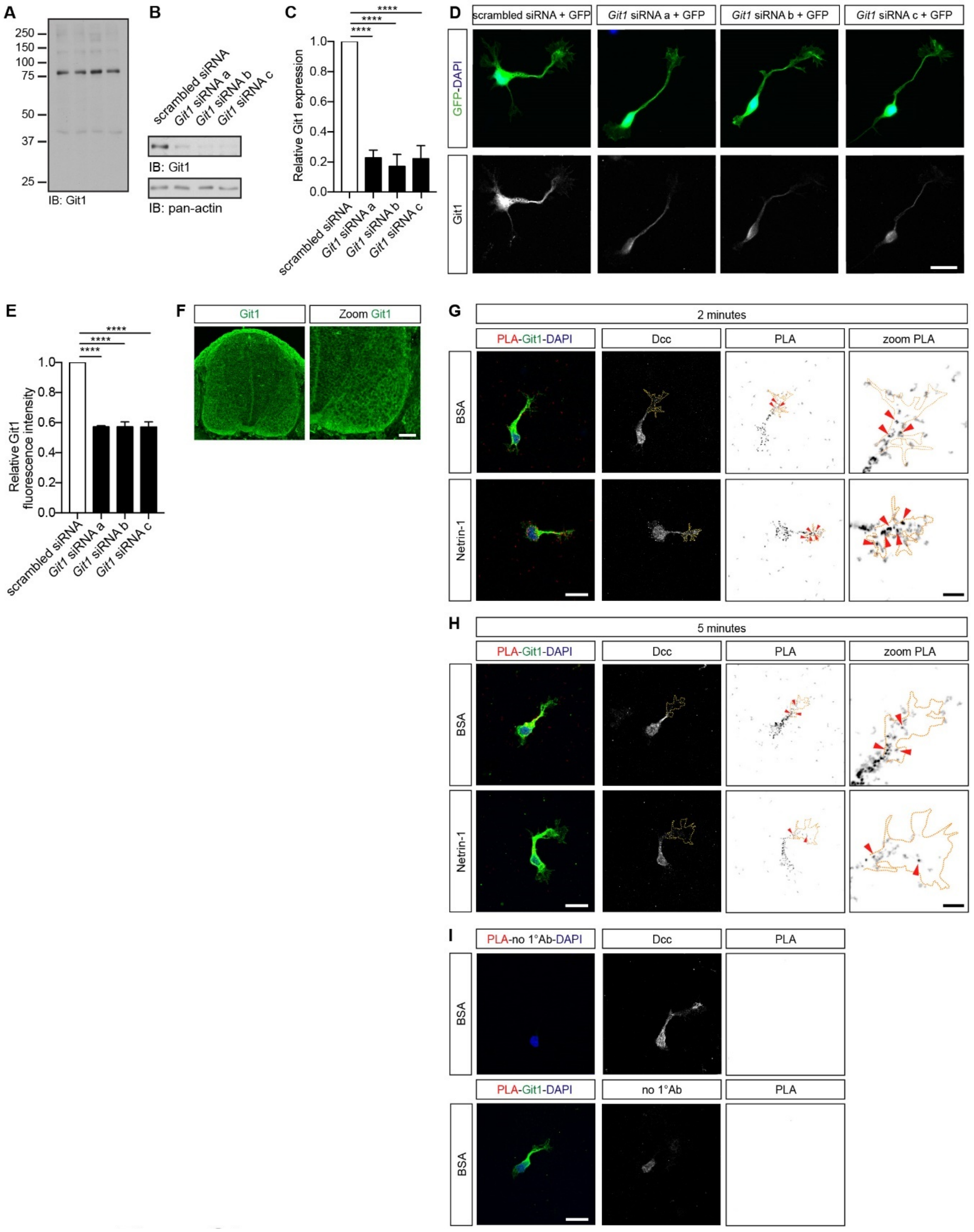

Fig. S3. Git1 interacts with Dcc in growth cones, and this interaction is modulated by Netrin-1.

Related to Fig. 4.

(A) Dissociated commissural neurons were lysed and Git1 expression was analyzed by Western blotting. The Git1 antibody detects one major band on the Western blot at the expected

molecular weight for Git1. **(B)** Commissural neurons were transfected with scrambled siRNA, *Git1* siRNA (a, b or c), and Git1 expression was analyzed by Western blotting. **(C)** The mean (±SEM) intensity of Git1 signal. *Git1* siRNA efficiently knocked down expression of Git1 as assessed by Western blotting. n = 4 experiments. One-way ANOVA, Bonferroni's multiple comparisons test **** p<0.0001. **(D)** Commissural neurons were transfected with scrambled siRNA, *Git1* siRNA (a, b or c), fixed and immunostained for Git1 and GFP. Scale bar: 20 µm. **(E)** The mean (±SEM) intensity of the Git1 immunofluorescence signal. n = 4 experiments, 15 growth cones per condition, per experiment. One-way ANOVA, Bonferroni's multiple comparisons test **** p<0.0001. **(F)** Mouse E11.5 neural tube cross-sections were immunostained for Git1. Scale bar: zoom 25 µm. **(G, H)** Dissociated commissural neurons were treated with 0.1 µg/ml BSA or Netrin-1 for 2 and 5 min respectively, then fixed with 4% PFA. The PLA assay was performed for Dcc and Git1. After the PLA reaction, Dcc and Git1 were counter-stained for visualization. Scale bar: 20 µm (left), 7 µm (zoom). **(I)** Omission of one of the two primary antibodies to control for potential non-specific binding of the secondary antibodies and the quality of PLA probes. Data are representative of 6 independent experiments, 15 growth cones per condition, per experiment.

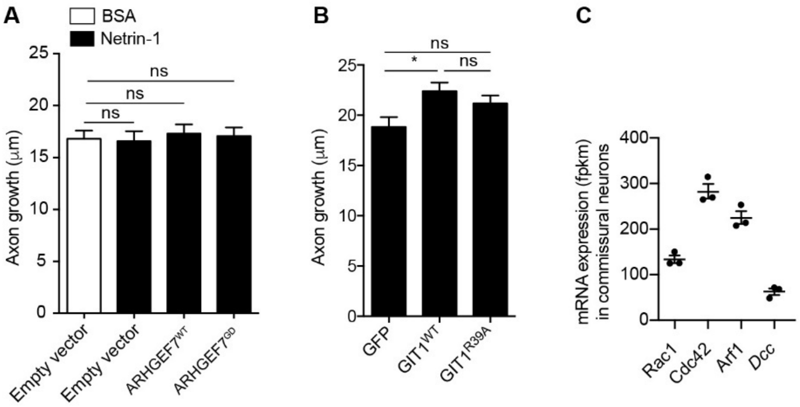

Fig. S4. Inhibition of Arhgef7 or Git1 activity has no effect on axon growth.
Related to Fig. 5.
(A) Commissural neurons were transfected with empty vector, $ARHGEF7^{WT}$ or $ARHGEF7^{GD}$ expression plasmids and then exposed to a gradient of Netrin-1 (0.1 µg/ml in the outer well) or BSA in the Dunn chamber. Axon growth (mean ± SEM) over 2 h. n=144, 134, 161, 166 respectively from 5 experiments. One-way ANOVA, Tukey's multiple comparisons test. **(B)** Commissural neurons were transfected with GFP, $Git1^{WT}$ and Git^{R39A} expression plasmids, and then exposed to a gradient of Netrin-1 (0.1 µg/ml in the outer well) in the Dunn chamber. Axon growth (mean ± SEM) over 2 h. n=125, 176, 234 respectively from 6 experiments. One-way ANOVA, Tukey's multiple comparisons test, *$p<0.05$. **(C)** The mean mRNA expression (± SEM) of *Rac1*, *Cdc42*, *Arf1* and *Dcc* in dissociated commissural neurons (n=3).

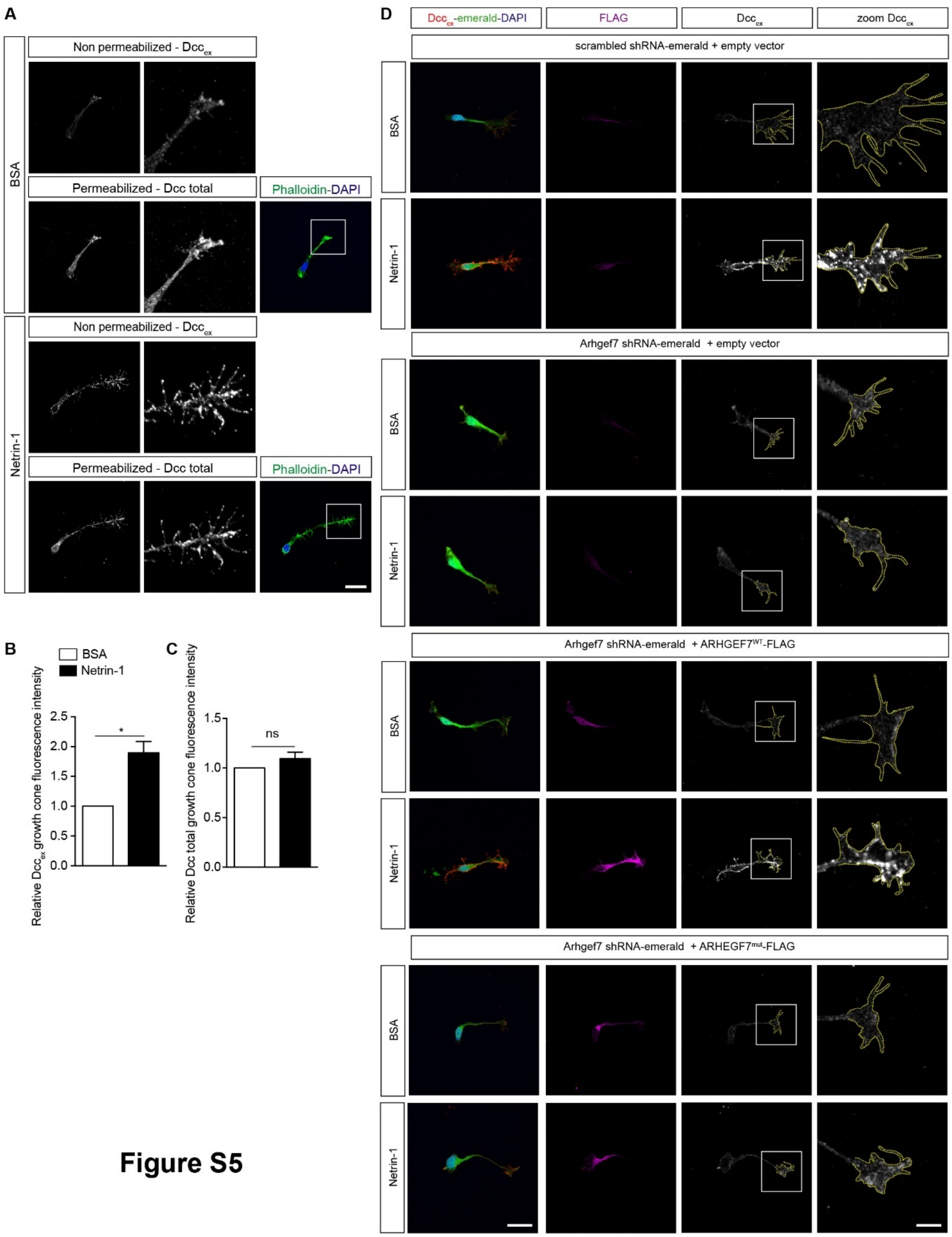

Fig. S5. Netrin-1 stimulation increases extracellular Dcc, but not total Dcc, and this requires Arhgef7.
Related to Fig. 6.
(A) Commissural neurons were fixed with 4% PFA and immunostained in the absence of Triton-X-100 with an anti-Dcc_{ex} antibody which recognizes the extracellular domain of Dcc (upper,

non-permeabilized) to detect cell surface Dcc. This was followed by permeabilization with Triton-X-100 and immunostaining with an anti-Dcc antibody that recognizes the intracellular domain in the presence of Triton X-100 (lower, permeabilized) to detect total Dcc. Scale bar, 20 µm. **(B, C)** Quantification of the relative extracellular Dcc growth cone fluorescence intensity and relative total Dcc growth cone fluorescence intensity. Netrin-1 stimulation increases extracellular Dcc at the growth cone but does not change the total amount of Dcc in the growth cone. n=4 experiments, 15 growth cones per condition, per experiment. Paired t test, *$p<0.05$. **(D)** Commissural neurons were transfected with scrambled shRNA, *Arhgef7* shRNA, empty vector, Arhgef7^{WT}-FLAG and Arhgef7^{mut}-FLAG expression vectors as indicated. Cells were stimulated with 0.1 µg/ml Netrin-1 or BSA for 5 min and fixed. Extracellular Dcc was detected by immunostaining under non-permeabilizing conditions. Emerald-GFP (which marks shRNA-transfected cells) and FLAG were detected by immunostaining under permeabilizing conditions. n=4 experiments, 15 growth cones per condition per experiment. Scale bar: 20 µm (left), 7 µm (zoom).

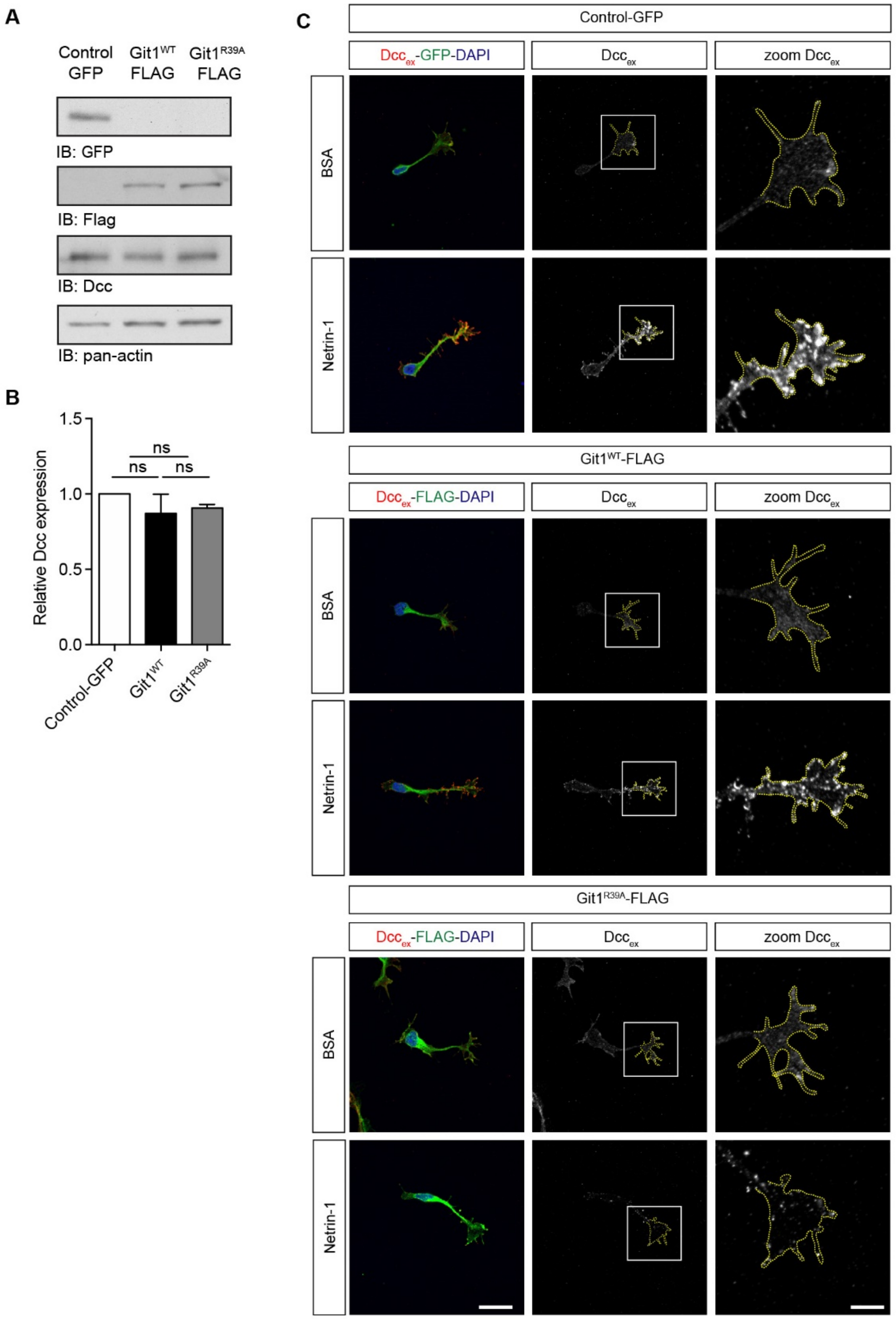

Fig. S6. Git activity is required for the Netrin-1-induced increase in cell surface Dcc but does not affect total Dcc levels.

Related to Fig. 6.

(A) Lysates from commissural neurons expressing control-GFP, Git1^{WT}-FLAG and Git1^{R39A}-FLAG were analyzed by Western blotting. Git1^{WT}-FLAG and Git1^{R39A}-FLAG expression had no effect on the amount of total Dcc. **(B)** The relative amount (mean ± SEM) of Dcc protein

normalized to the amount of pan-actin in the cell lysate. n=3, one-way ANOVA, Dunnett's multiple comparisons test. **(C)** Commissural neurons were transfected with control-GFP, $Git1^{WT}$-FLAG and $Git1^{R39A}$-FLAG expression vectors. Cells were stimulated for 5 minutes with 0.1 µg/ml Netrin-1 or BSA and fixed. Extracellular Dcc was detected by immunostaining under non-permeabilizing conditions. GFP and FLAG were detected by immunostaining under permeabilizing conditions. n=3 experiments, 15 growth cones per condition, per experiment.

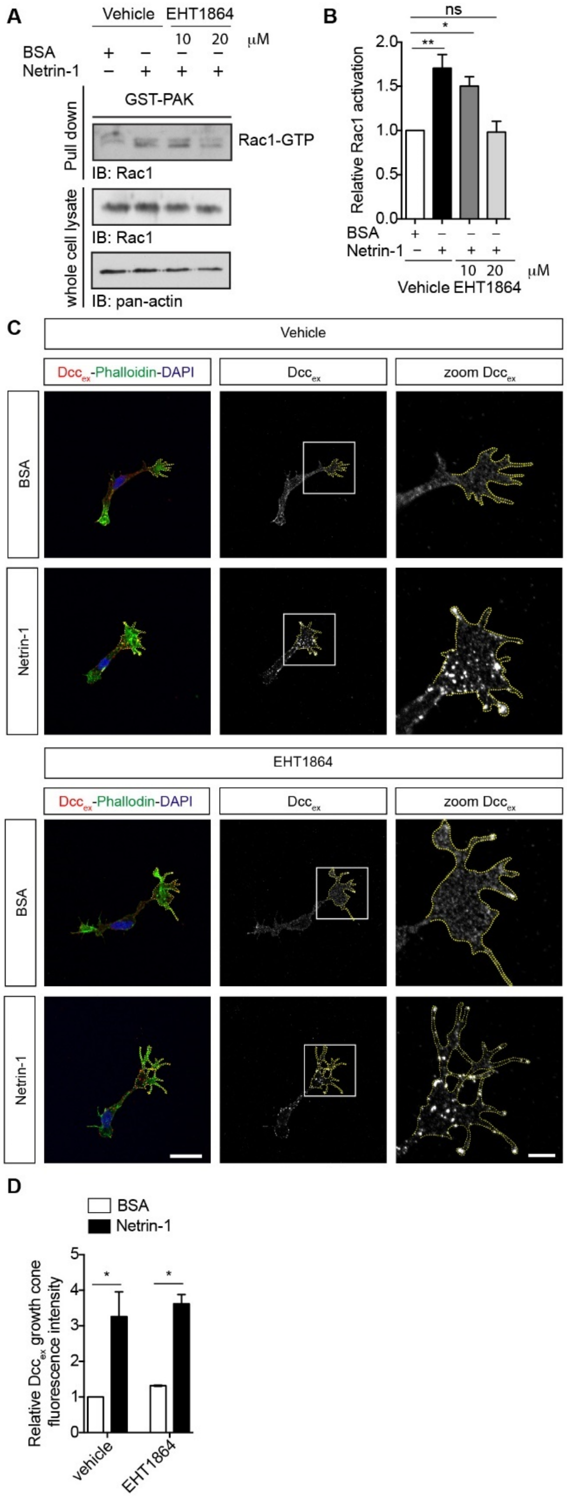

Fig. S7. Rac1 is not required for the Netrin-1-induced increase in cell surface Dcc.
Related to Fig. 6.
(A) Commissural neurons were treated for 2 h with vehicle or EHT1864 (10 or 20 µM) and stimulated for 5 minutes with 0.1 µg/ml Netrin-1 or BSA. Cells were lysed and assayed for Rac1

activation by pull-down of active Rac1 using GST-PAK-coated beads. The amount of Rac1 in pull-downs and in total cell lysates was detected by immunoblotting with the indicated antibodies. **(B)** Relative Rac1 activity (mean ± SEM) was normalized to the total amount of Rac1. 20 μM EHT1864 completely inhibited Netrin-1 induction of Rac1 activity. n=4, one-way ANOVA, Dunnett's multiple comparisons test, *p<0.05 **p<0.01. **(C)** Commissural neurons were treated with vehicle or EHT1864 (20 μM, 2 h) and were stimulated for 5 minutes with 0.1 μg/ml Netrin-1 or BSA and fixed. Extracellular Dcc was detected by immunostaining under non-permeabilizing conditions. F-actin was detected with phalloidin under permeabilizing conditions. n=3 experiments, 15 growth cones per condition, per experiment. **(D)** Commissural neurons were treated with vehicle or EHT1864 (20 μM, overnight) and were stimulated for 5 minutes with 0.1 μg/ml Netrin-1 or BSA and fixed. Extracellular Dcc was detected by immunostaining under non-permeabilizing conditions and the mean intensity (±SEM) of extracellular Dcc fluorescence in growth cones was quantified. n=3 experiments, 15 growth cones per condition, per experiment. Two-way ANOVA, Tukey's multiple comparisons test, *p<0.05. Overnight inhibition of Rac1 does not block the Netrin-1-induced increase in cell surface Dcc.

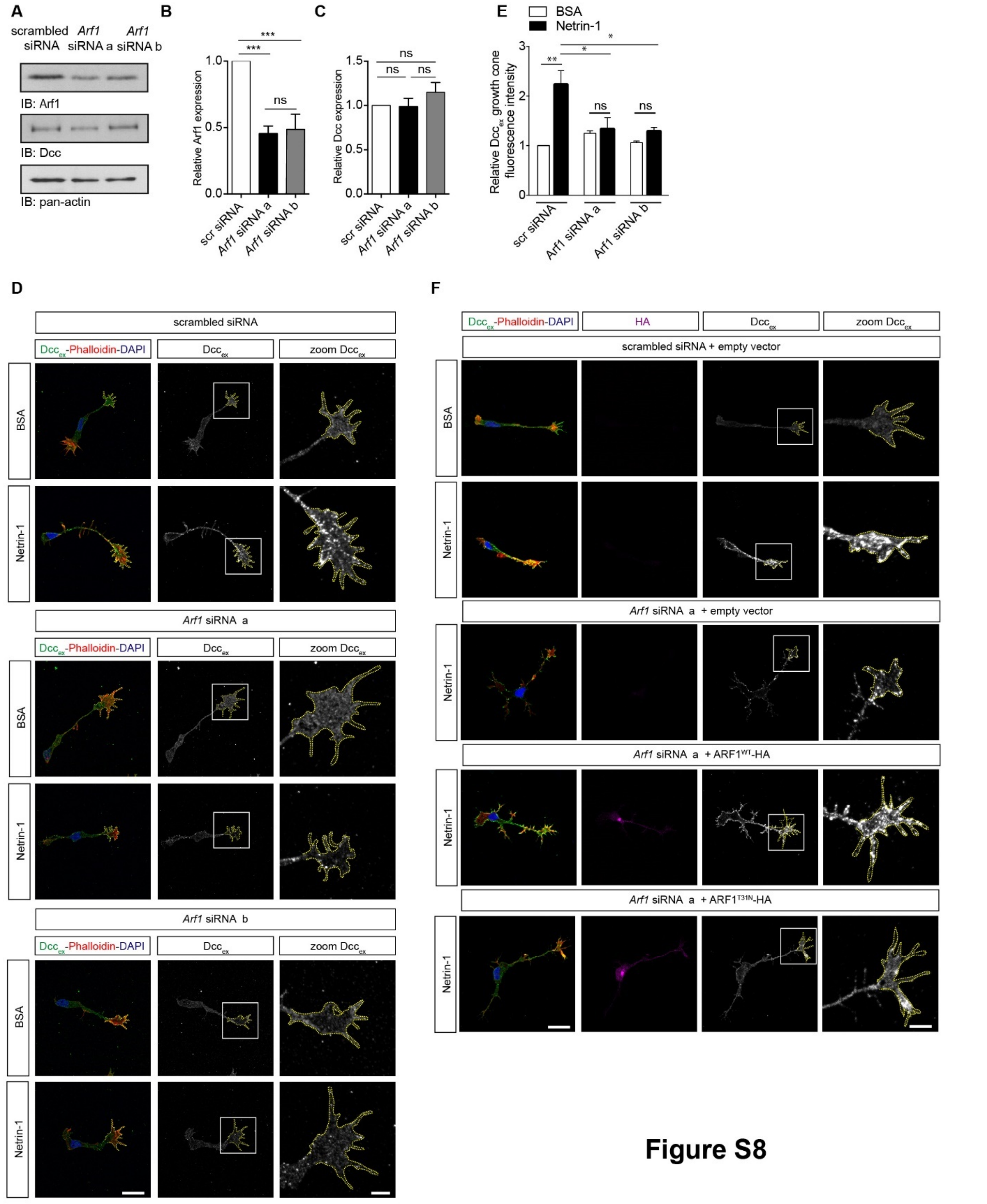

Figure S8

Fig. S8. Arf1 is required for the Netrin-1-induced increase in cell surface Dcc.
Related to Fig. 6.
(**A**) Commissural neurons were transfected with scrambled siRNA, *Arf1* siRNA (a or b) and protein levels were analyzed by Western blotting. **(B, C)** The relative amount (mean ± SEM) of

Arf1 and Dcc protein normalized to the amount of pan-actin in the cell lysate. *Arf1* siRNA reduced Arf1 protein levels, but had no effect on Dcc protein levels. n≥4, one-way ANOVA, Bonferroni's multiple comparisons test. ***p<0.001. **(D)** Commissural neurons were transfected with scrambled siRNA, *Arf1* siRNA (a or b) and were stimulated with 0.1 µg/ml Netrin-1 or BSA for 5 min and fixed. Extracellular Dcc was detected by immunostaining under non-permeabilizing conditions. F-actin was detected with phalloidin under permeabilizing conditions. n=3 experiments, 15 growth cones per condition, per experiment. **(E)** The mean intensity (±SEM) of extracellular Dcc fluorescence in growth cones. *Arf1* knockdown blocks the Netrin-1-induced increase in extracellular Dcc. n=3 experiments, 15 growth cones per condition, per experiment. Two-way Anova, Tukey's multiple comparisons test, *p<0.05, **p<0.01. **(F)** Commissural neurons were transfected with scrambled siRNA, *Arf1* siRNA a, empty vector, $ARF1^{WT}$-HA and $ARF1^{T31N}$-HA expression vectors as indicated. Cells were stimulated with 0.1 µg/ml Netrin-1 or BSA for 5 min and fixed. Extracellular Dcc was detected by immunostaining under non-permeabilizing conditions. HA and F-actin (using phalloidin) were detected under permeabilizing conditions. Scale bar: 20 µm.

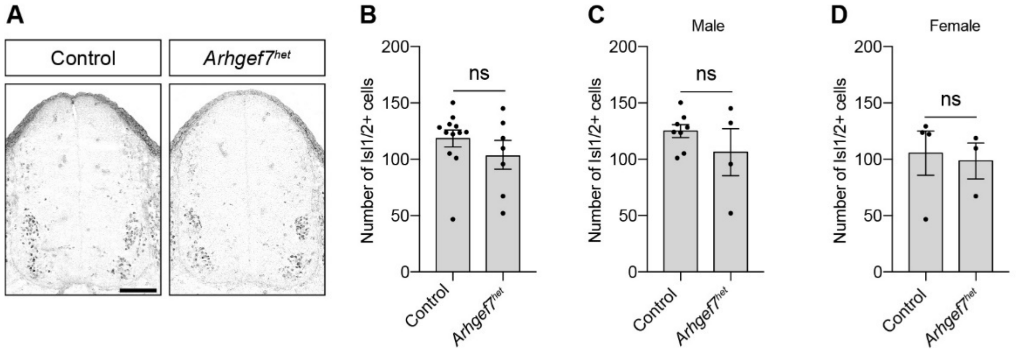

Fig. S9. ***Arhgef7***^{het} **mice have the same number of Isl1/2+ cells as control mice.**
Related to Fig. 7.
(A) Isl1/2 immunostaining of E11.5 spinal cord cross-sections of control and *Arhgef7*^{het} embryos. Scale bar: 100 µm. **(B-D)** Quantification of the number of Isl1/2+ cells present in the motor column area (mean ± SEM) for all embryos (B), males (C) and females (D). Control and *Arhgef7*^{het} mice have the same number of Isl1/2 cells present in the motor column area. Number of embryos: control n=12 (8 male, 4 female), *Arhgef7*^{het} n=7 (4 male, 3 female), 3 sections analyzed per embryo. Mann-Whitney test.
